# Dietary effects on the retina of hamsters

**DOI:** 10.1096/fj.202403390R

**Published:** 2025-03-18

**Authors:** Nicole El‐Darzi, Natalia Mast, Yong Li, Irina A. Pikuleva

**Affiliations:** ^1^ Department of Ophthalmology and Visual Sciences Case Western Reserve University Cleveland Ohio USA

**Keywords:** cholesterol, diet, hamster, HDL, indocyanine green, lipoprotein, retina

## Abstract

The retina is a sensory tissue in the back of the eye, which captures visual information and relays it to the brain. The retinal pigment epithelium separates the neural retina from the choroidal (systemic) circulation and is thereby exposed to circulating lipoprotein particles. Herein, we used hamsters and conducted various retinal evaluations of animals fed either a normal diet or a Western‐type diet (WTD). Prior to evaluations, hamsters were injected with indocyanine green (ICG), a fluorescent dye that binds to various proteins and lipids in the systemic circulation. The WTD increased plasma levels of total and HDL cholesterol 1.8‐ and 2.1‐fold, respectively, and led to additional HDL_2_ and HDL_3_ subpopulations. The diet also increased the ICG fluorescence in the retinal pigment epithelium and the underlying choroidal circulation on histological tracking and altered retinal protein abundance as assessed by proteomics. Functional enrichments were found in the retinal gene expression, energy production, intracellular transport, cytoskeleton‐ and synapse‐related processes, and protein ubiquitination. The biochemical basis linking the WTD, retinal energy production, and retinal neurotransmission was suggested as well. The data obtained were then compared with those from our previous investigations of hamsters and different mouse genotypes. We identified common retinal processes that can be affected by circulating lipoprotein particles regardless of the mechanism by which their levels and subpopulations were altered (through diet or genetic modification). Thus, we obtained novel mechanistic insights into how lipids in the systemic circulation can affect the retina.

## Graphical abstract

Western‐type diet increased in hamsters the levels of their blood total cholesterol (TC) and HDL as well as the heterogeneity of their blood HDL subpopulations HDL_2_ (yellow circles) and HDL_3_ (green circles). Western‐type diet also affected the expression of various retinal proteins and perhaps some retinal processes that are mediated by these proteins.

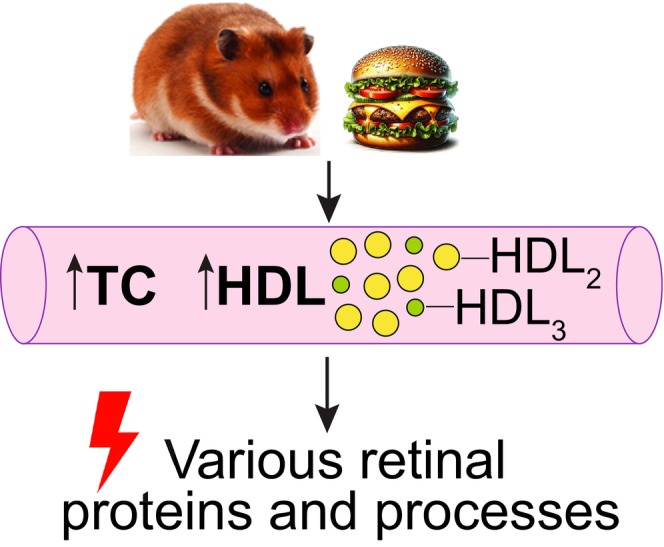

**Abbreviations**
AMD: age‐related macular degeneration
APOB100: apolipoprotein B100
APOC: apolipoprotein C
BrM: Bruch's membrane
CFD: cholesterol‐ and fat‐enriched diet
ChC: choroidal circulation
DEP: differentially expressed proteins
ETC: electron transport chain
ICG: indocyanine green
ICGA: indocyanine green angiography
LPPs: lipoprotein particle
ND: normal diet
NR: neural retina
RPE: retinal pigment epithelium
SyC: systemic circulation
TC: total cholesterol
TCA: tricarboxylic acid
WT: wild type
WTD: Western‐type diet
α‐KG: α‐ketoglutarate

## 1 INTRODUCTION

In mammals, cholesterol is constantly exchanged between different organs and cell types. This exchange is mostly mediated by lipoprotein particles (LPPs), which transport water‐insoluble cholesterol through the systemic circulation (SyC) and other body fluids.^1^ Of the circulating LPPs, low‐density LPPs or LDL deliver cholesterol to different organs, whereas high‐density LPPs or HDL mediate the so‐called reverse cholesterol transport or cholesterol removal from extrahepatic organs to the liver for metabolism into bile acids or excretion in bile or feces.^1^ HDL are heterogeneous and, depending on the isolation technique, may be classified based on their density, size, electrophoretic mobility, and composition.^1^, ^2^ Density ultracentrifugation, the golden standard approach for LPP separation, was introduced first and revealed two major HDL subclasses—HDL_2_ and HDL_3_‐isolated by the density ranges of 1.063–1.125 and 1.125–1.210 g/mL, respectively.^3^, ^4^ HDL_2_ are lighter and larger because they are more lipid‐rich (i.e., carry more cholesterol), whereas HDL_3_ are heavier and smaller because they are more protein‐rich and carry fewer lipids.^2^, ^5^, ^6^ It is still not clear whether HDL_2_ or HDL_3_ are more protective against cardiovascular disease. Yet, biochemical data suggest that functionally, HDL_3_ are superior to HDL_2_ in promoting cellular cholesterol efflux, protecting LDL from oxidation, and inhibiting thrombosis, inflammation, and apoptosis.^7^, ^8^

Whole‐body cholesterol homeostasis varies in different species, and among rodents, hamsters are closest to humans in terms of their plasma lipid profile, whole body cholesterol synthesis rate, bile acid metabolism, and dietary responses.^9^ This is because hamsters express cholesterol ester transfer protein, which mice do not express, and produce full‐length apolipoprotein B (APOB100) in the liver and the truncated APOB variant (APOB48) in the intestine, the latter being the only isoform produced in mice.^10^, ^11^, ^12^ In addition, hamsters have a relatively low rate of whole‐body cholesterol biosynthesis,^13^ do not upregulate their bile acid biosynthesis in response to dietary cholesterol,^14^, ^15^, ^16^ and take up the majority of LDL cholesterol via LDL receptors.^13^, ^17^ Therefore, the LDL/HDL ratios are 0.04 (2.2/51 mg/dL)–0.05 (2.2/46 mg/dL) in mice, 0.40 (15/38 mg/dL)–0.49 (27/55 mg/dL) in hamsters, and ~2.2 (100/45 mg/dL) in normolipidemic humans (Table 1),^19^, ^20^, ^21^ that is, much lower in mice than in humans, with hamsters being in the middle. Also, not only does the LDL/HDL ratio vary in different species, but also the relative HDL_2_ and HDL_3_ content: HDL_2_ is the predominant HDL subclass in mice (71%) and hamsters (66%) but is less abundant in humans (19% in men and 30% in women).^22^, ^23^

**TABLE 1:** Plasma and retinal cholesterol levels in different species.

Species	Diets^a^	Plasma					Retinal TC, nmol/mg protein	% of retinal cholesterol from blood
		TC, mg/dL	LDL, mg/dL	HDL, mg/dL	LDL/HDL	TG, mg/dL		
Humans^b^	WTD	<150	<100	>45	2.2	<150	nm^c^	nm
Hamsters^d^	ND	78 ± 31	15 ± 11	38 ± 10	0.40	88 ± 57	nm	nm
Hamsters^d^	WTD	141 ± 25*	17 ± 2	81 ± 19**	0.21	158 ± 161	nm	nm
Hamsters^e^	ND	112 ± 16	27 ± 7	55 ± 10	0.49	96 ± 39	88 ± 7	
Hamsters^e^	CFD1	240 ± 25***	46 ± 15***	121 ± 19***	0.38	374 ± 182***	113 ± 9***	47%
C57BL/6J mice^d^	ND	100 ± 15	2.2 ± 0.4	51 ± 10	0.04	94 ± 24	nm	nm
C57BL/6J mice^d^	WTD	143 ± 60	5.8 ± 2.2**	66 ± 26	0.09	89 ± 37	nm	nm
C57BL/6J mice^f^	ND	105 ± 5	2.2 ± 0.4	46 ± 5	0.05	75 ± 35	51 ± 4	nm
C57BL/6J mice^f^	CFD2	96 ± 16	3.4 ± 1.3	53 ± 11	0.06	89 ± 19	52 ± 3	21%
APOB100 mice^f^	ND	121 ± 11	14 ± 3.7	41 ± 5	0.34	125 ± 5	46 ± 2	nm
APOB100 mice^f^	CFD2	160 ± 27*	nm	nm	nm	nm	44 ± 2	23%

*Note*: All data represent the mean ± SD of the measurements in individual animals (*n* = 5–14 per group). Only statistical significance between the same study groups is indicated. Please see Figures S1 and S2 for statistical analyses based on diet and animal species.

^a^ Normal diet (Prolab Isopro RMH 3000) contained 0.02% (w/w) cholesterol and 5.4% (w/w) fat. WTD (Western‐type diet, TD.88137 Envigo) contained 0.15% (w/w) cholesterol and 21% (w/w) milk fat. Hamsters were fed WTD for 7 weeks, and mice were fed this diet for 10 weeks. CFD1 (cholesterol‐ and fat‐enriched custom‐made diet 1) contained 0.15% (w/w) cholesterol and 10% (w/w) fat, of which 5% was from peanut oil. Hamsters were fed CFD1 for 2 weeks. CFD2 (cholesterol‐ and fat‐enriched custom‐made diet 2) contained 0.3% (w/w) cholesterol and 15% (w/w) fat, of which 10% was from peanut oil. Mice were fed CFD2 for 2 weeks.

^b^ Lipid profile recommended by the American Heart Association.^18^

^c^ nm, not measured.

^d^ Data from the present work; 6‐month‐old male hamsters and 4.5‐month‐old male mice.

^e^ Data from Ref. [19]; 6‐month‐old male hamsters.

^f^ Data from Ref. [20]; 12‐month‐old male mice.

**FIGURE 1:**
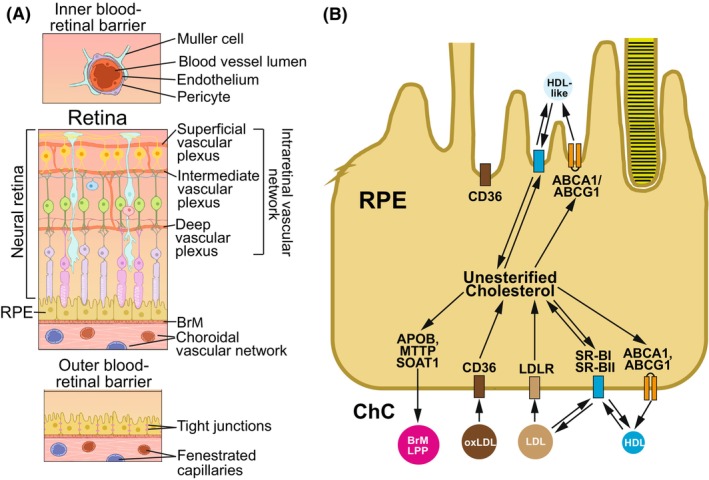
The neural retina, retinal pigment epithelium (RPE), and vascular networks. (A) Schematic representation of the retina comprised of the neural retina and RPE and served by two vascular networks, intraretinal and choroidal. The endothelial cells of the intraretinal vascular network are linked by tight junctions, which along with Muller cells, astrocytes, and pericytes establish the inner blood–retinal barrier that prevents passage of plasma proteins and other macromolecules in or out of the blood vessels. The outer blood‐retinal barrier is formed by the endothelial cells of the choroidal vascular network, by Bruch's membrane, and by the RPE cells. The endothelial cells of the choroidal vasculature are fenestrated and supply the retina with nutrients, water, and ions, while removing waste material. Bruch's membrane allows passive diffusion of various molecules, and the RPE cells form tight junctions but have different receptors on its basal side to take up certain molecules. Thus, the outer blood‐retinal barrier plays an essential role in retinal physiology by mediating a bi‐directional molecules' exchange between the retina and choroidal circulation (ChC).^24^ (B) Schematic representation of an RPE cell showing the location of the receptors for different LPP particles.^26^, ^28^, ^33^, ^45^ The ABCA1 and ABCG1 transporters that efflux cholesterol to HDL are also shown.^28^, ^30^, ^45^, ^46^, ^47^, ^48^, ^49^, ^50^, ^51^ BrM‐LPP, Bruch's membrane lipoprotein particles^35^, ^36^, ^37^, ^38^; oxLDl, oxidized LDL. Panel A for this figure was licensed from Carlson Stock Art and is used with permission; panel B was taken from^20^ and modified.

The retina is a multicellular and multilayered organ composed of the neural retina (NR) and the underlying retinal pigment epithelium (RPE) (Figure 1A). The retina is served by two independent vascular networks, intraretinal and choroidal, and is separated from the SyC by the inner and outer blood‐retinal barriers.^24^ The inner blood‐retinal barrier prevents cholesterol exchange between the intraretinal vasculature and NR (Figure 1A). The outer blood‐retinal barrier separates the NR from the choroidal circulation (ChC) and includes the RPE, positioned between the two structures (Figure 1A). Cholesterol from the ChC can reach the NR because the RPE can take up plasma LPP via different receptors (LDLR, SR‐BI, SR‐BII, and CD36, Figure 1B) present on its basal side.^25^, ^26^, ^27^, ^28^, ^29^, ^30^, ^31^, ^32^, ^33^ In mice and hamsters, this uptake accounts for ~21% and 47% of total retinal cholesterol input, respectively, despite the LDL/HDL ratio in the two species being 8‐ to 2‐fold different.^19^, ^34^ In part, this is because the RPE can produce unique APOB‐containing LPPs called Bruch's membrane LPP (BrM‐LPP, Figure 1B), which cycle the blood‐borne cholesterol back to the systemic (choroidal) circulation.^35^, ^36^, ^37^, ^38^ Yet, BrM‐LPP accumulate with age in BrM^39^ and are the components of soft drusen and basal linear deposits,^40^, ^41^, ^42^ the cholesterol‐rich pathognomonic lesions for age‐related macular degeneration (AMD),^43^ a major cause of legal blindness in the elderly.^44^

There are no consistent correlations between plasma LPP and AMD, although genetic variants of the HDL‐related genes (*CETP*, *LIPC*, *APOE*, and *ABCA1*) are risk factors for this disease.^21^ Therefore, it was suggested that the link could be between different plasma HDL subclasses, which could vary in their AMD risk‐conferring properties.^21^, ^52^ Hence, in the present work, we conducted plasma LPP and retinal characterizations of hamsters fed a Western‐type diet (WTD) and compared the data obtained with our previous characterizations of hamsters and mice to begin to assess the WTD‐fed hamsters as a model for studies of retinal responses to changes in serum LPP.

## 2 MATERIALS AND METHODS

### 2.1 Reagents

Regular rodent chow or normal diet (ND, 5P75‐5P76 Prolab Isopro RMH 3000) was from LabDiet (Saxonburg, PA, USA) and contained 0.02% cholesterol (w/w) and 5.4% fat (w/w). WTD was from ENVIGO (now Inotiv, West Lafayette, IN, USA, TD.88137) and contained 0.15% cholesterol (w/w) and 21% fat (w/w). ND versus WTD were also different in the content of antioxidants, vitamins, and various fatty acids including essential fatty acids (linoleic acid: 1.73% vs. 2.3%, w/w; and linolenic acid: 0.16% vs. 0.7%, w/w). Indocyanine green (ICG) was from Diagnostic Green (Farmington Hills, MI, USA, NDC 70100‐424‐01). All other chemicals were from MilliporeSigma (St. Louis, MO, USA).

### 2.2 Animals

Golden Syrian hamsters were from Charles River (Wilmington, MA, strain code: 049). Only male animals were used; as previously reported, no sex‐based differences were found in the hamster serum lipid profile and retinal content of different sterols.^53^ All hamsters were on ND until the age of 4.5 months, when the animals were randomly split, and a half were placed on WTD until the age of 6 months, that is, for 7 weeks. C57BL/6J mice were from the Jackson Laboratory (Bar Harbor, ME, USA, #000664) and were free from the retinal degeneration mutations, including *Crbl* ^*rd8*^. Only male mice were used to match male hamsters. Half of the mice were put on WTD from 1.5‐ to 4.5 months of age, that is, for 10 weeks. At the end of the dietary treatment, both hamsters and mice were mature (6 and 4.5 months of age, respectively), and their retinal cholesterol homeostasis was at a steady state, which is reached at ≥6 months in hamsters and ≥3 months in mice.^53^, ^54^, ^55^ Animal assignment to experimental groups was random and utilized the pool of all available animals; the sample size was based on previous experience. The investigators were not blinded with respect to treatment, as they were involved in both animal treatment and subsequent tissue isolation. Animals were maintained on a standard 12‐h light (~10 lux) dark cycle, with food and water provided ad libitum. All terminal experiments were conducted between 9 and 11 a.m. to avoid circadian rhythm effects. All animal experiments were approved by Case Western Reserve University's IACUC and conformed to the recommendations of the American Veterinary Association Panel on Euthanasia (protocols 2014‐0154 and 2020–0029).

### 2.3 ICG angiography (ICGA)

ICGA is usually used to assess the status of chorioretinal vasculature^56^, ^57^, ^58^ as its emission maximum is in the far‐red region (~832 nm), enabling imaging through the blood and ocular pigments.^56^, ^57^, ^58^ In addition, ICGA can also visualize lipid accumulations in BrM, which impede ICG passage from the ChC into the RPE and are presented as hypofluorescent spots in late phase ICGA.^59^, ^60^, ^61^, ^62^, ^63^, ^64^ Conversely, the fundus fluorescence is homogeneous in the late ICGA phase if the dye crosses BrM and enters the RPE.^60^, ^64^, ^65^, ^66^ Two weeks prior to the end of the dietary treatment, hamsters underwent ICGA as described.^20^, ^53^ Briefly, hamsters were anesthetized via intraperitoneal injections of 160 mg/kg ketamine and 7.5 mg/kg xylazine (Patterson Veterinary, Greeley, CO, USA, 07‐890‐8598 and 07‐808‐1947, respectively), and then were given a bolus intraperitoneal injection of ICG (4 mg/kg of body weight) or sterile water. Fundus images were acquired by a scanning laser ophthalmoscope (Spectralis HRA, Heidelberg Engineering, Franklin, MA, USA) at an early, intermediate, and late angiography phase with the laser beam being focused either on the retinal or choroidal vasculature. Animals were kept warm on heating pads and remained surgically sedated throughout the experiment. Subcutaneous injections of aqueous atipamezole, 0.75 mg/kg (Patterson Veterinary, 07‐867‐7097) were used to wake hamsters up.

### 2.4 Blood collection, processing, and plasma content

At the end of dietary treatments, food and bedding were removed from both hamsters and mice, and hamster cheek pouches were emptied with a cotton swab.^53^ All animals were fasted overnight for 12 h. The next morning, hamsters were injected intraperitoneally with ICG (4 mg/kg of body weight) or sterile water, and 15 min post‐injection were deeply anesthetized with a 200 mg/kg ketamine and 10 mg/kg xylazine bolus injection. Blood was withdrawn 17 min post‐injection via cardiac puncture in EDTA‐coated tubes, and hamsters were decapitated by guillotine followed by eye enucleation. Mice were deeply sedated with an 80 mg/kg ketamine and 7 mg/kg xylazine bolus injection, and their blood was withdrawn via cardiac puncture in EDTA‐coated tubes. To obtain plasma, the blood from both species was processed as described^20^ and analyzed for lipid and albumin content by IDEXX Laboratories (North Grafton, MA, USA). In a separate experiment (data are not shown), we established that in vivo ICG injection does not affect the quantifications of plasma LPP.

### 2.5 Plasma LPP isolation

Plasma LPP were isolated by density ultracentrifugation as described.^20^ Briefly, individual plasma samples (*n* = 3/diet, each ~1.1 mL) were centrifuged at 15 000 *g* and 4°C for 30 min to remove platelets and chylomicrons.^67^ Samples from the same diet were then pooled into a 3 mL volume, and their density was adjusted to 1.063 g/mL by KBr (Fisher Scientific, Hampton, NH, USA, S80134). Samples were placed into ultracentrifuge tubes, carefully overlayed with 30 mL of a cold solution of KBr of the same density in phosphate‐buffered saline (PBS), and subjected to the 1st ultracentrifugation in the bucket rotor at 100 000 *g* and 10°C for 24 h. The tube content was then carefully removed in 1.0‐mL aliquots from the tube top and measured for ICG fluorescence at 832 nm (excitation at 720 nm) and light scattering of LPP at 520 nm. Fractions containing each LPP type (LDL or HDL) were combined, and the density of the HDL‐containing fractions was adjusted to 1.125 g/mL by KBr. These fractions (2.7 mL for ND and 4.7 mL for a WTD) were placed into ultracentrifuge tubes and overlayed with cold KBr solution of the same density (27.3 mL for ND and 25.3 mL for a WTD). Samples were subjected to the 2nd ultracentrifugation (100 000 *g* at 10°C for 24 h) followed by the tube content removal in 1.0 mL aliquots and analyses for ICG fluorescence at 832 nm and light scattering at 520 nm. The density of the HDL_3_‐containing fractions (3.0 mL for ND and 4.5 mL for a WTD) was adjusted to 1.210 g/mL by KBr, and these fractions were placed in ultracentrifuge tubes. Samples were overlayed with cold KBr solution of the same density (27 mL for ND and 25.5 mL for a WTD) and were subjected to the 3rd ultracentrifugation (100 000 *g* and 10°C for 24 h). One‐milliliter aliquots were collected, analyzed, and pooled, and the identified HDL_2_ and HDL_3_ subpopulations were dialyzed against 10 mM Tris HCl buffer, pH 7.4, overnight at 4^o^C in the 3500 MWCO Slide‐A‐Lyzer Dialysis Cassettes (Thermo Fisher Scientific, #66330). These subpopulations were then concentrated using the 3K MWCO Pierce Concentrator (Thermo Fisher Scientific, #88512) and analyzed by SDS‐PAGE and Western blotting.

SDS‐PAGE (1 μg of protein per lane) and Western blotting (0.3 and 1 μg of protein per lane for detection of serum albumin and isoforms of apolipoprotein C, APOC, respectively) were carried out as described^20^ using 4%–15% Tris/Glycine gels (Bio‐Rad, Hercules, CA, USA). After separation, proteins were either visualized by the Silver Stain Kit (ThermoFisher Scientific Inc., Waltham, MA, USA, #24612) according to the manufacturer's instructions or transferred to the nitrocellulose membranes (Li‐Cor Biosciences, Lincoln, NE, USA, P/N 926‐31092). Membranes were then placed in the Odyssey blocking buffer (Li‐Cor Biosciences, #927‐40000) for 1.5 h at room temperature, followed by incubations with primary antibody overnight at 4°C and with secondary antibody for 1 h at room temperature in the Odyssey blocking buffer containing 0.1% Tween‐20. Rabbit polyclonal antibody to mouse serum albumin (Abcam, Cambridge, #ab34807, 1:1000 dilution), rabbit polyclonal antibody to mouse APOC1 (Invitrogen, #PA5102480, 1:1000 dilution), rabbit polyclonal antibody to mouse APOC3 (Invitrogen, #PA5116572, 1:1000 dilution), and purified goat anti‐rabbit antibody conjugated to IRDye 800 CW (Li‐Cor, Lincoln, #926‐68075, 1:25 000 dilution) were used. The intensity of fluorescent bands on the nitrocellulose membranes was quantified by an Odyssey infrared imaging system (Li‐Cor Biosciences).

### 2.6 Histology ICG tracking

One eye from each hamster was fixed for 15 min at room temperature in 20% dimethylsulfoxide (Fisher Scientific, Hampton, NH, USA, BP231‐100) containing 2% paraformaldehyde. Eyes were then embedded in the Tissue Tek O.C.T. compound (Sakura Finetek USA, Inc., Torrance, CA, USA #4583) and flash frozen in liquid nitrogen. Retinal sections were cut at a 15 μm thickness and imaged on a Zeiss Axio Scan.Z1 slide scanner (Carl Zeiss Microscopy, LLC, White Plains, NY, USA) equipped with a Colibri 7 far‐red LED light source and a Plan‐Apochromat 20×/0.8‐NA objective. The acquisition software was Zen Blue 3.1. For DAPI visualization, the filter illumination wavelength was 370–400 nm and the emission wavelength was 412–438 nm. For ICG visualization, the filter excitation wavelength was 720–750 nm and the filter emission wavelength was 769–807 nm. Deconvolved images were generated within Huygens Essential 23.10 software (Scientific Volume Imaging, Hilversum, the Netherlands) using the Standard deconvolution profile in Deconvolution Express and represented a maximum intensity projection of the deconvolved *z*‐stack. For the fluorescence intensity quantification, Metamorph Imaging Software (Molecular Devices, Downington, PA, USA) was used. Two retinal cross‐sections from one hamster eye were randomly selected and an identical threshold was applied to all images, including the control (no ICG injection). Ten equal‐sized regions were placed at random intervals along the RPE; their intensity values were recorded as relative fluorescent units and averaged for each hamster.

### 2.7 Retinal proteomics

One eye from each hamster was used for retina (NR+RPE) isolation as described previously for mice.^68^ These samples (each containing both NR and RPE) were then sent to Creative Proteomics (Shirley, NY, USA) for relative protein quantifications by the label‐free approach. Retinal proteomics was carried out as described^20^ using five retinas per diet from five hamsters. Briefly, retinal samples were solubilized in a lysis buffer, centrifuged, and the supernatant was collected. Samples were then separated by 12% SDS‐PAGE and stained with Coomassie Brilliant Blue. Gel slices were cut, placed in microcentrifuge tubes, and incubated with a 50 mM ammonium bicarbonate–acetonitrile mixture (1/1, v/v) to remove the dye. Proteins were then reduced with 10 mM dithiothreitol, alkylated with 50 mM iodoacetamide, and digested with trypsin (Promega, Madison, WI, USA, #VA9000). The trypsinolysis was quenched with 0.1% trifluoroacetic acid, and gel slices were removed by centrifugation. The supernatants obtained were lyophilized and reconstituted in 0.1% (v/v) formic acid in the LC–MS/MS grade water (MilliporeSigma #1.15333). Peptide concentrations were quantified by the optical density measurement at 280 nm and normalized to 0.1 μg/μL.

Samples (1 μg of protein) were run on an Ultimate 3000 nano UHPLC system (ThermoFisher Scientific, Inc.) coupled to a Q Exactive HF mass spectrometer (ThermoFisher Scientific, Inc.). The mass spectrometer was operated in the data‐dependent acquisition mode. Raw data files were processed with the MaxQuant software (Max Planck Institute of Biochemistry, Martinsried, Germany, version 1.6.2.6), and tandem mass spectra were searched against the UniProt hamster, mouse, and human databases. Proteins were quantified and normalized using MaxLFQ with a label‐free quantification minimum ratio of 1.2. Protein intensity values were normalized within the median. Differentially expressed proteins (DEP) were determined by one‐way ANOVA at a false discovery rate value of ≤.05. Proteins with non‐significant changes (*p* > .05) in abundance between the groups were excluded from the subsequent analysis, as were the proteins with less than a 1.2‐fold change in the relative abundance, even if this change was significant. DEPs were analyzed for functional enrichment by the Panther (version 19, https://www.pantherdb.org) and STRING (version 12.0, https://string‐db.org) online tools.^68^, ^69^ Venn diagrams and heatmaps were generated by an online tool (http://bioinformatics.psb.ugent.be/webtools/Venn/) and by the GraphPad Prism software (version 10, GraphPad Software, La Jolla, CA).

### 2.8 Statistics

All data were used, and apparent outliers were not excluded. Data represent the mean ± SD of the measurements in individual hamsters; the number of animals (*n*) is indicated in each figure. Data were analyzed by one‐way ANOVA with Tukey's multiple comparison test or a two‐tailed, unpaired Student's *t*‐test using GraphPad Prism software. Statistical significance was defined as *p ≤ .05, **p ≤ .01, and ***p ≤ .001. In histology comparisons of dietary treatments, retinal regions were matched by location.

## 3 RESULTS

### 3.1 ICGA

**FIGURE 2:**
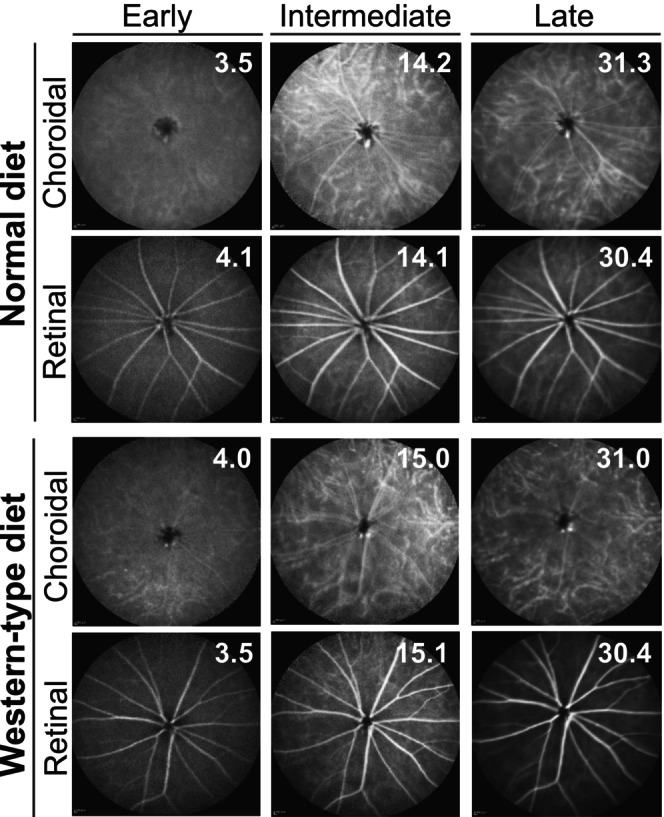
In vivo imaging of hamster retina by ICGA. Representative images (*n* = 5) of an early, intermediate and late‐stage fundus ICG fluorescence with the laser beam being focused either on the choroidal or retinal vascular networks. White numbers in the upper right corner indicate the post‐ICG injection time in minutes.

In vivo imaging by ICGA was used first to assess the dietary treatment effect on retinal vascular networks and lipid retention in BrM. Neither WTD nor ND‐fed hamsters had any apparent vascular abnormalities on ICGA (Figure 2), except the margins of the choriocapillaris, which were diffused in both groups, indicating increased vessel permeability. This feature was not observed in mice^20^ but was present in our previous evaluation of hamsters by fluorescein angiography.^53^ The only potential difference between the dietary groups was perhaps the more homogenous fluorescent background in the ND‐group than in the WTD group during the intermediate and late ICGA phases. This could be an indicator of possible ICG retention in BrM of the WTD group due to lipid accumulation. Yet, the intermediate and late ICGA fluorescence pattern in hamsters was quite different from those in humans,^59^, ^62^, ^63^, ^64^ making a definite conclusion difficult.

### 3.2 Plasma lipid profile

**FIGURE 3:**
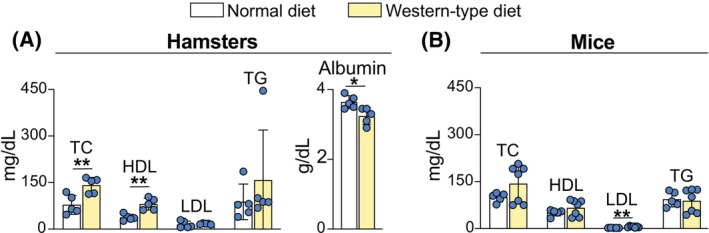
Plasma lipid profiles in hamsters (A) and mice (B) on different diets. Data were analyzed by a two‐tailed unpaired Student's *t*‐test (*n* = 5–7 animals per group); dots represent the measurements in individual animals. *p ≤ .05; **p ≤ .01.

The WTD effect on plasma lipids was evaluated at the end of dietary treatment after hamsters were injected with ICG for the 2nd time (2 weeks after the 1st ICG injection) and 17 min post‐injection had terminal blood collection followed by eye enucleation. As compared with ND, WTD significantly increased the levels of total cholesterol (TC, 1.8‐fold, 141 vs. 78 mg/dL, Figure 3A) with most of this increase (68%) being due to an increase in HDL cholesterol (2.1‐fold, 81 vs. 38 mg/dL). The levels of LDL cholesterol were the same in the two dietary groups (17 vs. 15 mg/dL) as were the levels of triglycerides (158 vs. 88 mg/dL). The plasma albumin content was decreased in the WTD‐ versus ND‐group but only moderately (1.13‐fold, 3.2 vs. 3.6 g/dL). Albumin is a protein, not a lipid; nevertheless, it is important to document changes in its serum levels as they may affect cholesterol offload from the RPE to serum LPP. Indeed, albumin was shown to facilitate cellular efflux of unesterified cholesterol to extracellular acceptors such as HDL and LDL, and like serum HDL, to be inversely associated with cardiovascular disease.^69^, ^70^, ^71^, ^72^, ^73^, ^74^

The WTD effect on plasma lipids in mice was evaluated as well, but after a longer dietary treatment time (10 weeks for mice vs. 7 weeks for hamsters) as mice are known to be more resistant to dietary‐induced atherosclerosis.^9^, ^75^ Nevertheless, no significant changes were observed in the WTD‐ versus ND‐group in the plasma levels of TC (143 vs. 100 mg/dL) and HDL cholesterol (66 vs. 51 mg/dL) as well as triglycerides (89 vs. 94 mg/dL) (Figure 3B). Only the levels of LDL cholesterol were increased 2.6‐fold (from 5.8 vs. 2.2 mg/dL) but still remained very low. Thus, the principal changes between the WTD‐fed hamsters versus WTD‐fed mice were increases in the former in plasma total and HDL cholesterol and the higher absolute LDL values.

### 3.3 HDL heterogeneity

**FIGURE 4:**
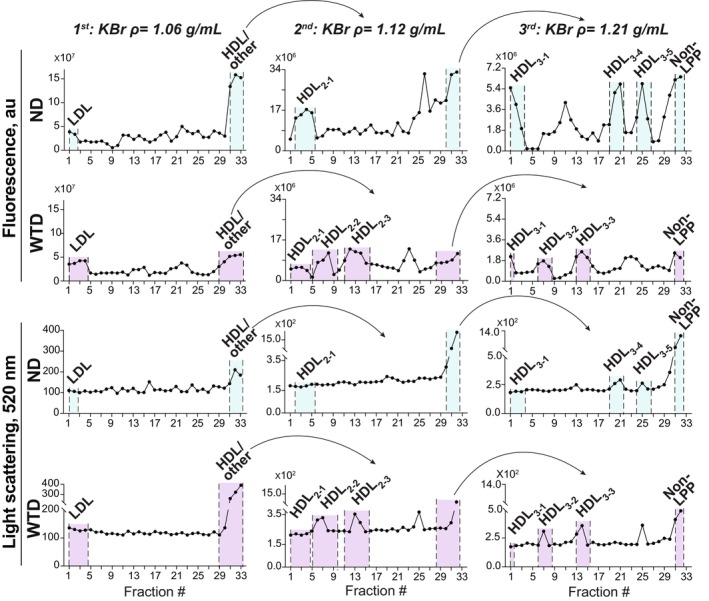
Plasma LPP isolation by three KBr density ultracentrifugations. After each ultracentrifugation, 1‐mL fractions were collected from the tube top to the tube bottom and their fluorescence and light scattering were measured. Fraction pooling for hamsters on normal diet (ND) and western‐type diet (WTD) is indicated by the cyan and lavender highlights, respectively.

Injection of ICG to live mice before blood collection enabled the characterization of the WTD effects on HDL subclasses as anionic ICG binds to polar phospholipids on LPP but does not interact with the LPP neutral lipids, such as esterified cholesterol, unesterified cholesterol, and triglycerides.^76^, ^77^, ^78^, ^79^, ^80^ Hence, plasma LPP were subjected to three KBr density ultracentrifugations to separate LDL from HDL and HDL_2_ from HDL_3_,^2^, ^23^ which were then identified based on their flotation, ICG fluorescence, and light scattering, the approach which we developed previously.^20^ These density ultracentrifugations documented that both dietary groups had the canonically floating HDL_2_ and HDL_3_, which we designated as the subpopulations HDL_2‐1_ and HDL_3‐1_, respectively (Figure 4). In addition, both groups had the HDL subpopulations of higher density as indicated by the fractions in which the peaks of ICG fluorescence and light scattering overlapped (Figure 4). For HDL_2_, these higher density subpopulations were only present in the WTD group and were numbered as HDL_2‐2_ and HDL_2‐3_ to reflect their increasing density. For HDL_3_, these additional subpopulations were present in both groups but had different densities—lower in the WTD group (HDL_3‐2_ and HDL_3‐3_) and higher in the ND group (HDL_3‐4_ and HDL_3‐5_). Thus, in hamsters, the WTD increased the HDL_2_ heterogeneity and altered the subpopulations of HDL_3_.

**FIGURE 5:**
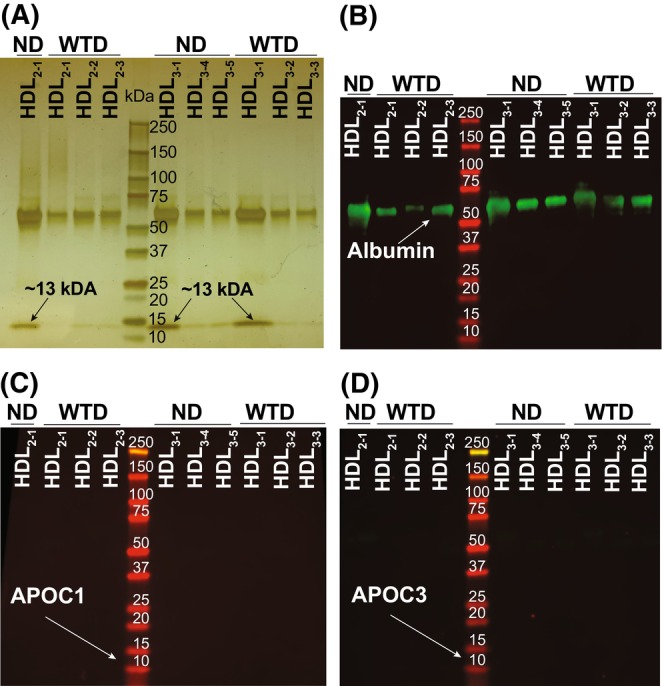
SDS‐PAGE and Western blot analyses of the HDL subpopulations isolated by density ultracentrifugations from hamster plasma. The same amount of protein (1 μg) from each HDL subpopulation was applied per each lane for SDS‐PAGE (A). For Western blots (B–D), the protein amount per lane was 0.3 μg for detection of serum albumin and 1 μg for detection of the isoforms of apolipoprotein C (APOC). The molecular weight standards were run in the middle gel lane. ND, normal diet; WTD, Western‐type diet.

In addition to lipids, ICG binds to various plasma proteins.^77^, ^78^, ^79^, ^80^ Therefore, the isolated HDL subpopulations were analyzed by SDS‐PAGE and then by Western blotting (Figure 5). All subpopulations had a prominent band at ~68 kDa, which corresponded to the molecular weight of hamster serum albumin and was particularly prominent in the subpopulations of ND HDL_2‐1_ and HDL_3‐1_ and in WTD HDL_3‐1_ (Figure 5A). These three HDL subpopulations also had a prominent band at ~13 kDa, corresponding to the molecular weight of the hamster APOC isoforms.^23^ Hence, Western blotting was carried out next to detect serum albumin and the APOC isoforms using the antibodies against the corresponding mouse proteins, as antibodies against hamster proteins were not available. Western blotting confirmed the albumin presence in the 68 kDa band (Figure 5B) and showed that the immunofluorescence intensity was the highest in the subpopulations of ND HDL_2‐1_, ND HDL_3‐1_, and WTD HDL_3‐1_, in which the 68 kDa band was also the most abundant on SDS‐PAGE. Yet, the APOC1 and APOC3 isoforms were not detected in the ~13 kDa band (Figure 5C,D), possibly due to a lack of antibody specificity to hamster proteins. Thus, serum albumin was detected in all HDL subpopulations, likely as a contaminant, and apparently was labeled with ICG,^77^, ^79^, ^81^ thus explaining the low light scattering of the subpopulations of ND HDL_2‐1_, ND HDL_3‐1_, and WTD HDL_3‐1_, yet their high fluorescence intensity.

### 3.4 Histology ICG tracking

**FIGURE 6:**
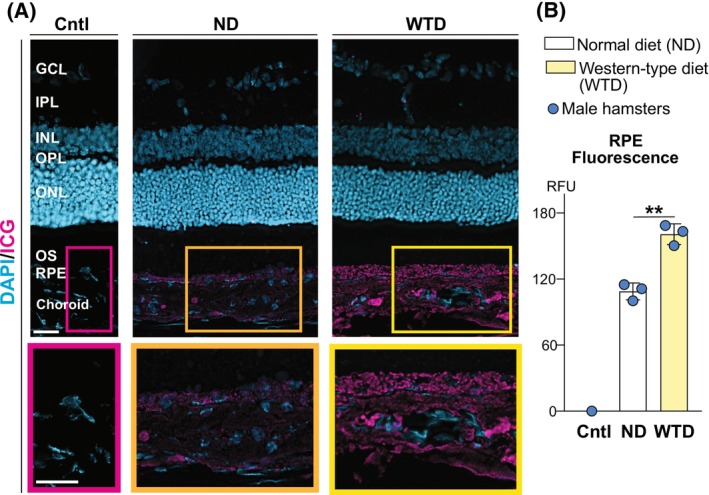
Histological ICG tracking. Representative retinal cross‐sections from the eyes of hamsters on normal diet (ND) and Western‐type diet (WTD) (*n* = 3 animals per diet) 17 min post‐intraperitoneal ICG injection. Control hamster (Cntl) was injected with sterile water. Color boxes indicate enlarged chorioretinal regions shown at the bottom. The quantification of ICG fluorescence in relative fluorescence units (RFU) is also shown (B). Scale bars 25 μm. GCL, the ganglion cell layer; IPL, the inner plexiform layer; INL, the inner nuclear layer; OPL, the outer plexiform layer; ONL, the outer nuclear layer; OS, the photoreceptor outer segments; RPE, retinal pigment epithelium. **p ≤ .01.

Previously, we demonstrated that not only LPP in the plasma but also in the retina can be tracked after an in vivo ICG injection. This can be done histologically after the retina of the ICG‐injected animals is sectioned, and retinal cross sections are analyzed for ICG fluorescence.^20^ Indeed, the ICG fluorescence was detected in both dietary groups and was much more pronounced in the WTD‐ than in the ND‐fed hamsters (Figure 6). In both groups, the fluorescence was mainly localized to the chorio‐RPE region and did not extend into the subretinal space, an indication that the plasma lipid content was mainly processed in the RPE. In the RPE, the ICG fluorescence was punctate, suggestive of LPP and/or vesicle labeling as vesicular transport is required to transit excess proteins and other cargo through cells.^82^ In addition, several ICG fluorescent dots were also detected inside some of the retinal blood vessels but not in the NR, consistent with the presence of the inner blood‐retinal barrier. Thus, the histology ICG tracking revealed that the WTD‐induced increases in the levels of plasma TC and HDL cholesterol mainly affected the chorio‐RPE region, and that the RPE had increased plasma content localized either on LPP and/or vesicles.

### 3.5 Retinal proteomics

**FIGURE 7:**
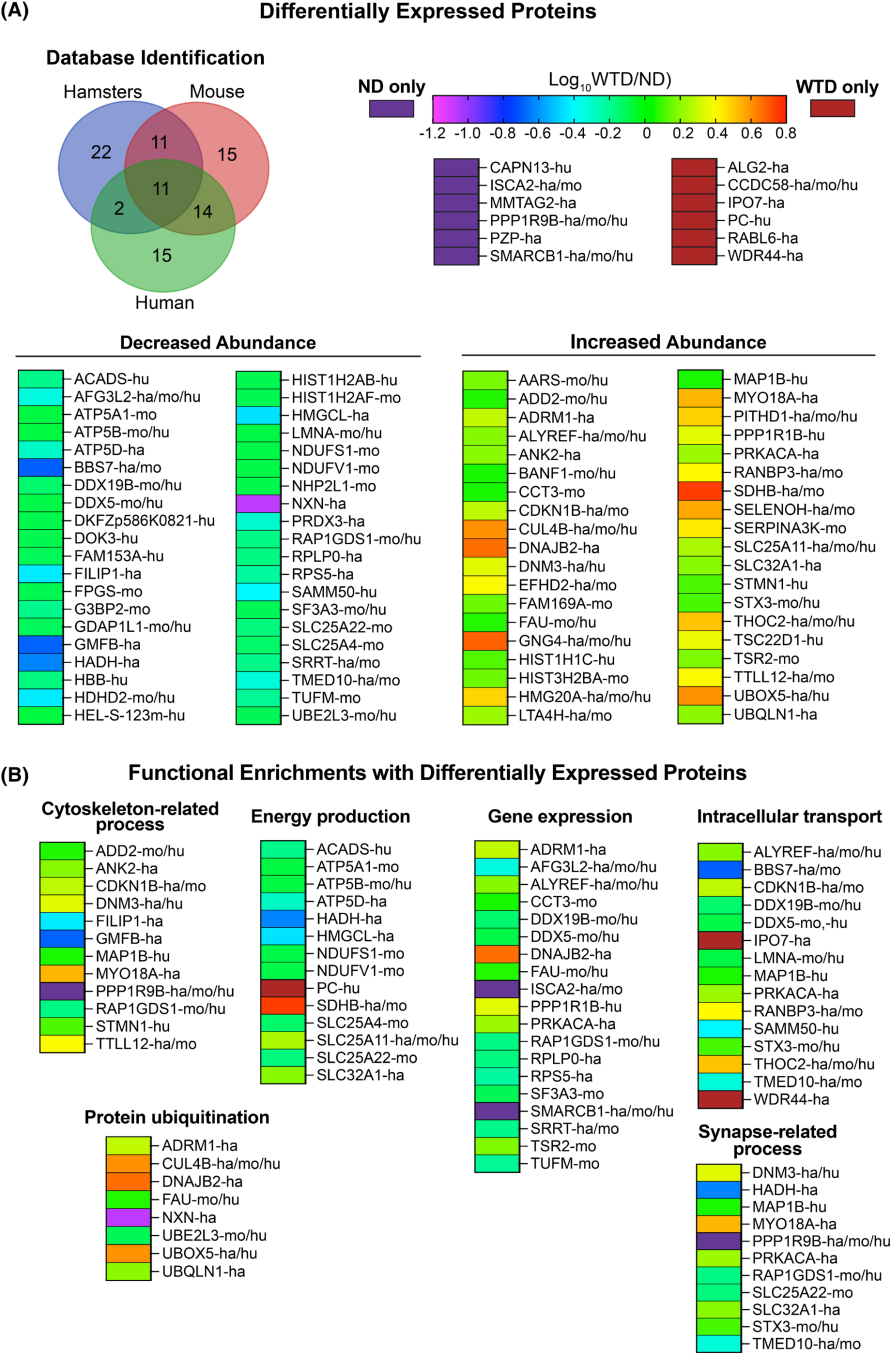
Retinal proteomics of hamsters on western‐type diet (WTD) versus normal diet (ND). (A) The identification of differentially expressed proteins in different databases and changes in the protein abundance. (B) Biological processes enriched with differentially expressed proteins. ha, hu, and mo indicate the identification of differentially expressed proteins in hamster, human, and mouse databases, respectively. See main text for details.

The label‐free approach was used to compare retinal protein abundance. Proteins were identified by searches against the hamster (23 887 entries), mouse (54 707 entries), and human (82 861 entries) databases, as the latter two have more entries than the former. A total of 90 DEPs were identified: 11 were common between the three databases, and the remaining were unique either to the hamster database (22 DEPs), mouse database (15 DEPs), human database (15 DEPs) or overlapped between the databases (Figure 7A). Further, of the total 90 DEPs identified, 46 and 44 had decreased and increased abundance, respectively, in the WTD‐ versus ND‐groups. Of them, 6 DEPs were only found in the ND‐group (CAPN13, ISCA2, MMTAG2, PPP1R9B, PZP, and SMARCB1) and 6 DEPs were only found in the WTD group (ALG2, CCDC58, IPO7, PC, RABL6, and WDR44) (Figure 7A). All DEPs were analyzed for functional enrichment by the Panther online tool, which identified six major general processes that could be affected by changes in protein abundance (Figure 7B). These were gene expression (19 DEPs), energy production (14 DEPS), intracellular transport (14 DEPs), cytoskeleton‐related processes (12 DEPs), synapse‐related (11 DEPs), and protein ubiquitination (8 DEPs). Of these processes, the intracellular non‐neurotransmitter vesicular transport was represented by 5 DEPs (DNM3, MAP1B, MYO18A, TMED10, and WDR44, the latter being found only in the WTD‐fed hamsters). All functionally enriched processes could be required to deal with the diet‐induced increases in plasma lipid and protein content delivered to the RPE on the LPP. Hence, the data obtained could represent meaningful mechanistic insights into the retinal homeostatic response to the WTD treatment.

## 4 DISCUSSION

This work is a continuation of our efforts to investigate the effect of plasma LPP profile on the retina. Previously, we characterized hamsters as well as mice, C57BL/6J (WT) and APOB100 transgenic (APOB100), fed ND and diets with different cholesterol and fat enrichments (CFD1 and CFD2).^19^, ^20^, ^53^ Herein, we started to characterize hamsters and WT mice fed WTD, a different CFD type, which had a higher amount of fat (a total of 21%, w/w) and extent of fat saturation (61%) as compared with CFD1 and CFD2 (Table 1). The present work enabled a comparison of all the data obtained so far and provided novel insights into how changes in the plasma LPP profile due to diet, interspecies differences, and genetic modification could affect the HDL subclass and subpopulation distribution, chorioretinal LPP trafficking, retinal cholesterol input, retinal content of cholesterol, and retinal proteome, as discussed below.

Indeed, WTD was investigated in the present work, and CFD1 was studied previously.^19^ Both diets were enriched with the same percentage of cholesterol (0.15%, w/w) but different amounts and sources of fat (21% from milk fat and 10% from mixed fat, w/w, respectively) (Table 1) and fatty acids. Both diets increased the levels of hamster plasma TC and HDL cholesterol, and either did not change (WTD) or increased (CFD1) hamster LDL cholesterol levels (Figure 3A, Table 1, Figure S1). As a result, the LDL/HDL ratio decreased in hamsters on both diets, yet retinal cholesterol content, which was previously determined in hamsters on CFD1 versus ND, was increased (Table 1), likely because 47% of this content was obtained via cholesterol uptake from the SyC.

In WT mice, the dietary treatments were with WTD in the present work and with CFD2 in our previous investigation^20^ (Table 1). As compared with CFD1, CFD2 had increases in both cholesterol content (from 0.15% to 0.3%, w/w) and fat content (from 10% to 15%, Table 1) as mice are known to be resistant to diet‐induced atherosclerosis.^9^, ^75^ In contrast to hamsters, neither WTD nor CFD2 increased in WT mice their within‐the‐study levels of plasma TC and HDL cholesterol, while the LDL cholesterol was either increased (on WTD) or unchanged (on CFD2) (Table 1). The LDL cholesterol was still very low in WT mice on both diets (e.g., 5.8 mg/dL on WTD) as compared with the HDL cholesterol (e.g., 66 mg/dL on WTD, Table 1); therefore, the diets increased the LDL/HDL ratio only up to 2.3‐fold (0.09 vs. 0.04, WTD vs. ND, respectively, Table 1). The retinal cholesterol content was only measured in WT mice on CFD2 and was unaltered versus the ND‐group, consistent with the lack of changes in the plasma lipid profile in the CFD2 group, with 21% of this content being obtained from the SyC (Table 1).

APOB100 is the major apolipoprotein in LDL.^1^ Previously, we characterized APOB100 mice fed ND versus WT mice fed ND.^20^ The LDL cholesterol levels were increased in the ND‐fed APOB100 versus WT mice more than sixfold (14 vs. 2.2 mg/dL, Table 1, Figure S2) and became comparable with those in hamsters. This LDL increase also led to a 6.8‐fold increase, as compared with WT mice on ND, in the LDL/HDL ratio (0.34 vs. 0.05, Table 1) as the HDL cholesterol content remained unchanged in APOB100 on ND (41 vs. 46 mg/dL, Table 1, Figure S2). Yet, the levels of plasma total cholesterol in APOB100 versus WT mice on ND were the same and were the levels of retinal cholesterol.^20^ Putting APOB100 mice on CFD2 further increased, relative to APOB100 mice on ND, the levels of their plasma TC but still did not change the levels of their retinal cholesterol (Table 1). Retinal uptake of cholesterol from the SyC was also not changed in APOB100 versus WT on CFD2 (23% vs. 21%, Table 1). Thus, a paradigm is beginning to emerge that the retinal content of cholesterol and retinal cholesterol uptake from the SyC may not be significantly affected at normal or modestly elevated plasma TC levels (like in APOB100 mice on CFD2, Table 1) and the LDL/HDL ratios of up to 0.34 (like in APOB100 mice on ND). Yet, at similar LDL/HDL ratios but a larger increase in plasma TC (like in hamsters on CFD1, Table 1), there could be a change (increase) in both retinal cholesterol content and the SyC contribution to this content. Further studies are required to confirm our findings.

Our assessments by density ultracentrifugations and histological tracking of the ICG‐labeled LPP provided additional important insights. Specifically, in hamsters on WTD versus ND, not only was the plasma HDL content increased (Figure 3) but also the heterogeneity of the HDL_2_ subclass (Figure 4). In addition, two HDL_3_ subpopulations became less dense (Figure 4). Conversely, APOB100 versus WT mice on ND studied previously^20^ had similar HDL content. Yet, the APOB100 Tg expression altered the ratio between the HDL subclasses by increasing the content of the higher density HDL_3_, thus making the HDL_2_/HDL_3_ ratio more similar to that in humans.^20^ Thus, both dietary treatment of hamsters and the genetic modification of mice could be used to study the effect on the retina of different plasma LPP, including different subclasses and subpopulations of HDL.

LDL and HDL bind to different receptors (LDLR and SRBI, respectively, Figure 1B), which use different mechanisms to process LPP content. LDLR endocytoses LDL followed by LDL degradation in lysosomes and lipid release in the cytoplasm.^1^ SRBI do not internalize HDL; rather, they mediate bi‐directional (by gradient) cholesterol exchange between HDL and cells.^1^, ^83^ In addition, SRBI transport other lipids into cells.^1^, ^83^ Consistent with this SRBI mechanism and HDL increase in hamsters on WTD versus ND, the former had ICG fluorescence mostly confined to the chorio‐RPE space, which was punctate in the RPE (Figure 6), likely reflecting the RPE homeostatic response to higher amounts of lipids that were delivered to this layer on HDL from the ChC. Similarly, an increase in the RPE ICG fluorescence was also observed in APOB100 versus WT mice on ND, which we studied previously.^20^ Yet, the difference with hamsters on WTD was that besides the RPE, the ICG fluorescence in the ND‐fed APOB100 versus WT mice was also detected around the blood vessels of the ChC, and there was a continuous track of fluorescent dots connecting these blood vessels and the RPE.^20^ We explained this fluorescent pattern with an increased LPP cycling between the RPE and ChC and suggested that this is a protective mechanism against increased plasma TC and LDL load in the SyC.^20^ Overall, in both species, hamsters on WTD versus ND and in APOB100 mice on ND versus WT mice on ND, the ICG histological tracking clearly demonstrated that the RPE protects, at least in part, the NR from increased load of LPP from the SyC as most of the ICG fluorescence was observed in this layer (Figure 6).

**FIGURE 8:**
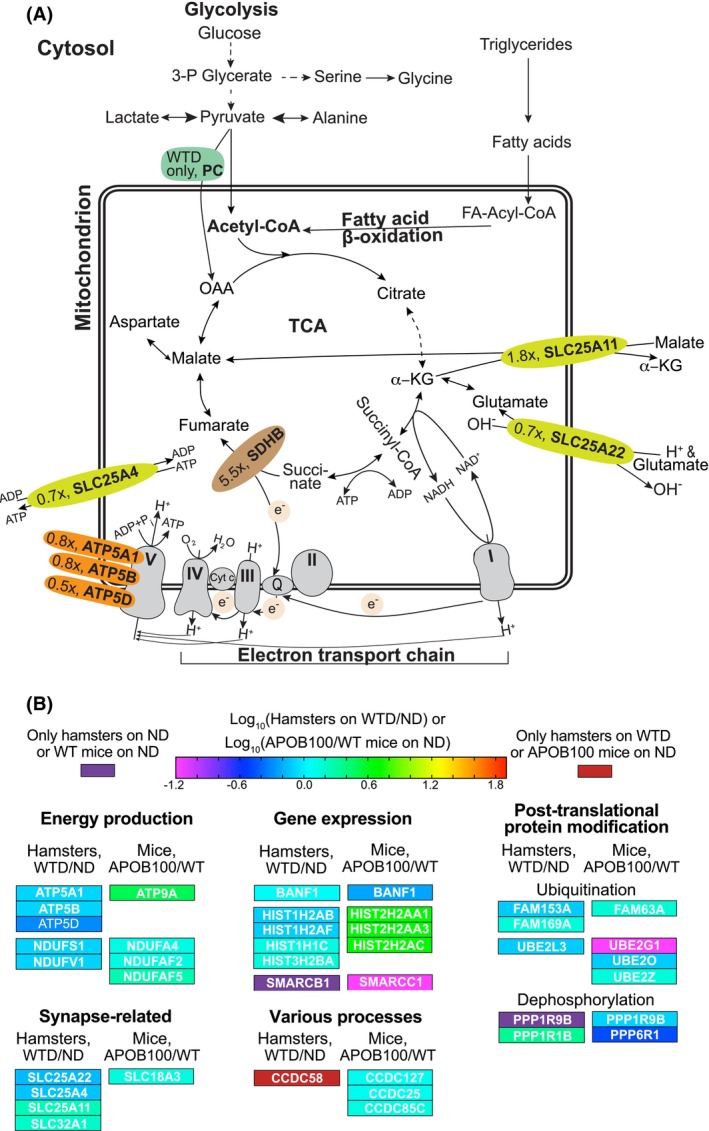
Proteomics analyses. (A) Some of the enzymatic reactions catalyzed by differentially expressed proteins. x, a fold change in protein abundance in hamsters on WTD versus ND. PC catalyzes the irreversible conversion of pyruvate into oxaloacetate, OAA.^84^ SLC25A11 exports α‐KG from mitochondria into the cytosol in exchange for malate or other dicarboxylic acids.^85^ SLC25A22 imports glutamate and H^+^ from cytosol to mitochondria in exchange for OH^−^.^86^ SDHB is a dual function enzyme, catalyzing the oxidation of succinate to fumarate in the TCA cycle and also transferring electrons from succinate to ubiquinone (Q) in the mitochondrial electron transport chain.^87^ SLC25A4 imports ADP into the mitochondrial matrix for ATP synthesis, and exports ATP to fuel the cell.^88^, ^89^ ATP 5A1, 5B, and 5D are subunits of ATP synthase, which produces ATP from ADP and P_i_ (inorganic phosphate) using the energy generated by the electron transport chain (ETC).^90^ Dashed lines indicate multiple reactions. (B) Overlapping processes and protein families suggested by a comparison of differentially expressed proteins in hamsters on WTD versus ND with those in mice, APOB100 versus WT, on ND. The ATP family is represented by the subunits of ATP synthase in the ETC, which generates 90% of cellular ATP.^91^ The NDUF family members are the NADH–ubiquinone oxidoreductase/dehydrogenases from different electron transport chain complexes.^92^, ^93^, ^94^, ^95^ The BAN family is represented by BANF1 (barrier‐to‐autointegration factor 1), which plays fundamental roles in nuclear assembly, chromatin organization, and gene expression.^96^ The HIST family is various histones, that is, proteins that bind DNA and form chromatin.^97^ The SMARC family is the SWI/SNF‐related matrix‐associated Actin‐dependent regulators of chromatin.^98^ The FAM members are the deubiquinating enzymes.^99^ Conversely the UBE2 members are different ubiquitin‐conjugating enzymes E2.^100^ The PPP family are different subunits of phosphoprotein phosphatases^101^ with PPP1R9B, which overlapped in hamsters and mice, being a protein phosphatase 1 and Actin filament binding protein localized to dendritic spines.^102^ The SLC family is represented by different mitochondrial and vesicular amine or amino acid transporters.^103^ Finally, the members of the CCDC (coiled‐coil domain‐containing) protein family are involved in a wide variety of biological functions.^104^, ^105^

Unexpected insights were provided by retinal proteomics of hamsters on WTD versus ND (Figure 7A). This approach identified potentially affected processes that occur either in the NR (i.e., synapse‐related process) or could be operative in both the NR and RPE (all other processes in Figure 7B). The dietary effect on retinal energy production was particularly evident as retinal proteomics detected pyruvate carboxylase (PC) only in hamsters on WTD (Figure 8A), indicating a significant increase in enzyme abundance. PC catalyzes the irreversible conversion of pyruvate into oxaloacetate, which is then fluxed into the tricarboxylic acid (TCA) cycle and thereby is important for whole‐body energetics through regulation of gluconeogenesis in the liver, fatty acid synthesis in adipocytes, and insulin secretion in pancreatic β cells.^84^ In the retina, PC is known to be exclusively expressed in Müller cells, where it was shown to be required for de novo glutamate synthesis from α‐ketoglutarate (α‐KG) generated in the TCA cycle (Figure 8A), thus playing an important role in controlling the levels of glutamate, the major retinal excitatory neurotransmitter.^106^, ^107^ Thus, the present work suggested the biochemical basis linking WTD, retinal energy production, and retinal neurotransmission.

Remarkably, the retina of hamsters on WTD versus ND also had altered abundance of some proteins and enzymes that control the levels of the TCA cycle intermediates and determine the ATP synthase activity in the mitochondrial electron transport chain (ETC) (Figure 8A). Specifically, SLC25A11 (solute carrier family 25 member 11, 1.8‐fold change) and subunit B of succinate dehydrogenase (SDHB, 5.5‐fold change) had increased abundance, whereas SLC25A22 (solute carrier family 25 member 22, 0.7‐fold change), SLC25A4 (solute carrier family 25 member 4, 0.7‐fold change), and the subunits of ATP synthase or Complex V (ATP 5A1, 5B, and 5D, 0.8, 0.8, and 0.5‐fold change, respectively) had decreased abundance (see Figure 8A legend for the protein function description). Thus, it is feasible that feeding hamsters WTD rich in triglycerides increased their retinal fatty acid β‐oxidation and production of acetyl‐CoA (Figure 8A), an allosteric regulator of PC,^84^ which, along with increased PC expression, increased oxaloacetate flux in the TCA cycle. This increased oxaloacetate flux could in turn increase the α‐KG synthesis and SLC25A11 abundance and decrease the SLC25A22 abundance to normalize the α‐KG levels (Figure 8A). Further, it is possible that if these compensatory responses were not sufficient, there was a compensatory increase in SDHB abundance, although it is not clear whether this change would upregulate both of the enzyme activities. Probably not, as the abundance of the ATP synthase subunits and SLC25A4 exporting mitochondrial ATP was decreased in hamsters on WTD versus ND, which could be a reflection of selective upregulation of the SDHB‐mediated fumarate production at the expense of ubiquinone reduction. Overall, the proposed sequence of events is consistent with the knowledge that the retina needs energy to carry out phototransduction and neurotransmission and that this energy is derived from glucose and fatty acid β‐oxidation.^108^, ^109^, ^110^

Importantly, if WTD increased the flux of the intermediates through the TCA cycle and hence glutamate production, the latter can potentially lead to glutamate excitotoxicity. This inference is supported by the increased abundance of SLC32A1 (1.6‐fold, Figure 7A), an inhibitory amino acid transporter necessary for the uptake of GABA and glycine into the synaptic vesicles.^111^ Both GABA and glycine are involved in retinal neurotransmission and co‐localize with glutamate in a majority of cells stained for either of these two inhibitory neurotransmitters to be balanced by excitatory neurotransmission.^112^ Obviously, further studies are necessary to confirm our proposed effects of WTD on hamster retina and investigate the physiological consequences of these putative changes, which may enhance our understanding of how retinal lipid and glucose dyshomeostases contribute to neovascular AMD and other retinal diseases.^41^, ^110^, ^113^, ^114^, ^115^, ^116^

Finally, we compared changes in retinal proteomics in hamsters on WTD versus ND in the present work with those in APOB100 versus WT mice on ND in our previous study^20^ to gain insights into whether similar processes and proteins are affected by increases in plasma cholesterol and fat content as a result of increases in the levels of HDL and LDL, respectively (Table 1). Indeed, effects on similar general processes mediated by the members from the same protein family were found, although only two DEPs overlapped (BANF1 and PPP1R9B, Figure 8B). These processes (and protein families) included energy production (ATP and NDUF), gene expression (BANF, HIST, and SMARC), post‐translational protein modification (FAM and UBE from ubiquitination and PPP from dephosphorylation), and synapse‐related (SLC, see Figure 8B for the protein families' function). Thus, regardless of the mechanism by which circulating LPPs are enriched with cholesterol and fat (through diet or genetic modification), different rodent species can have similar effects on the retina mediated by the same families of proteins. It remains to be determined if our findings can be extrapolated to humans.

In summary, we studied the retinal effects of WTD on the hamster retina and compared the data obtained with those from our previous investigations of hamsters and different mouse genotypes. We found the conditions under which retinal cholesterol content and input can be increased by changes in the plasma lipid profile and identified other retinal processes that can potentially be affected by circulating LPP. Our data suggest that, depending on the study goals, both hamsters on WTD and APOB100 mice could be used as models for the evaluation of the plasma HDL and LDL effects, respectively, on the retina.

## AUTHOR CONTRIBUTIONS

Nicole El‐Darzi, Natalia Mast, and Irina A. Pikuleva conceived and designed the research; Nicole El‐Darzi, Natalia Mast, and Yong Li performed the research and acquired the data; Nicole El‐Darzi, Natalia Mast, and Irina A. Pikuleva analyzed and interpreted the data. All authors were involved in drafting and revising the manuscript.

## DISCLOSURES

The authors declare no conflicts of interest.

## Supporting information


Figure S1.

Figure S2.

## Data Availability

The data that support the findings of this study are available in the Materials and Methods and Results. The raw proteomics data that support the findings of this study will be shared upon request.

## References

[1] Feingold KR . Lipid and lipoprotein metabolism. Endocrinol Metab Clin N Am. 2022;51:437‐458. 10.1016/j.ecl.2022.02.008 35963623

[2] Kontush A , Lindahl M , Lhomme M , Calabresi L , Chapman MJ , Davidson WS . Structure of HDL: particle subclasses and molecular components. Handb Exp Pharmacol. 2015;224:3‐51. 25522985 10.1007/978-3-319-09665-0_1

[3] De Lalla OF , Gofman JW . Ultracentrifugal analysis of serum lipoproteins. Methods Biochem Anal. 1954;1:459‐478. 13193538 10.1002/9780470110171.ch16

[4] Chapman MJ . Comparative analysis of mammalian plasma lipoproteins. Methods Enzymol. 1986;128:70‐143. 3523143 10.1016/0076-6879(86)28063-5

[5] Patsch JR , Sailer S , Kostner G , Sandhofer F , Holasek A , Braunsteiner H . Separation of the main lipoprotein density classes from human plasma by rate‐zonal ultracentrifugation. J Lipid Res. 1974;15:356‐366. 4369164

[6] Kontush A , Chapman MJ . Functionally defective high‐density lipoprotein: a new therapeutic target at the crossroads of dyslipidemia, inflammation, and atherosclerosis. Pharmacol Rev. 2006;58:342‐374. 16968945 10.1124/pr.58.3.1

[7] Camont L , Lhomme M , Rached F , et al. Small, dense high‐density lipoprotein‐3 particles are enriched in negatively charged phospholipids: relevance to cellular cholesterol efflux, antioxidative, antithrombotic, anti‐inflammatory, and antiapoptotic functionalities. Arterioscler Thromb Vasc Biol. 2013;33:2715‐2723. 24092747 10.1161/ATVBAHA.113.301468

[8] Woudberg NJ , Pedretti S , Lecour S , et al. Pharmacological intervention to modulate HDL: what do we target? Front Pharmacol. 2018;8:989. doi:10.3389/fphar.2017.00989 29403378 PMC5786575

[9] Singh V , Tiwari RL , Dikshit M , Barthwal MK . Models to study atherosclerosis: a mechanistic insight. Curr Vasc Pharmacol. 2009;7:75‐109. 19149643 10.2174/157016109787354097

[10] Ha YC , Barter PJ . Differences in plasma cholesteryl ester transfer activity in sixteen vertebrate species. Comp Biochem Physiol B. 1982;71:265‐269. 7060347 10.1016/0305-0491(82)90252-8

[11] Greeve J , Altkemper I , Dieterich JH , Greten H , Windler E . Apolipoprotein B mRNA editing in 12 different mammalian species: hepatic expression is reflected in low concentrations of apoB‐containing plasma lipoproteins. J Lipid Res. 1993;34:1367‐1383. 8409768

[12] Reaves SK , Wu JY , Wu Y , et al. Regulation of intestinal apolipoprotein B mRNA editing levels by a zinc‐deficient diet and cDNA cloning of editing protein in hamsters. J Nutr. 2000;130:2166‐2173. 10958808 10.1093/jn/130.9.2166

[13] Dietschy JM , Turley SD . Control of cholesterol turnover in the mouse. J Biol Chem. 2002;277:3801‐3804. 11733542 10.1074/jbc.R100057200

[14] Dueland S , Drisko J , Graf L , Machleder D , Lusis AJ , Davis RA . Effect of dietary cholesterol and taurocholate on cholesterol 7 alpha‐hydroxylase and hepatic LDL receptors in inbred mice. J Lipid Res. 1993;34:923‐931. 8354958

[15] De Fabiani E , Crestani M , Marrapodi M , Pinelli A , Chiang JY , Galli G . Regulation of the hamster cholesterol 7 alpha‐hydroxylase gene (CYP7A): prevalence of negative over positive transcriptional control. Biochem Biophys Res Commun. 1996;226:663‐671. 8831673 10.1006/bbrc.1996.1412

[16] Chiang JYL , Kimmel R , Stroup D . Regulation of cholesterol 7α‐hydroxylase gene (CYP7A1) transcription by the liver orphan receptor (LXRα). Gene. 2001;262:257‐265. 11179691 10.1016/s0378-1119(00)00518-7

[17] Marsh JB , Welty FK , Lichtenstein AH , Lamon‐Fava S , Schaefer EJ . Apolipoprotein B metabolism in humans: studies with stable isotope‐labeled amino acid precursors. Atherosclerosis. 2002;162:227‐244. 11996942 10.1016/s0021-9150(01)00709-2

[18] Grundy SM , Stone NJ , Bailey AL , et al. 2018 AHA/ACC/AACVPR/AAPA/ABC/ACPM/ADA/AGS/APhA/ASPC/NLA/PCNA guideline on the management of blood cholesterol: a report of the American College of Cardiology/American Heart Association task force on clinical practice guidelines. Circulation. 2019;139:e1082‐e1143. 30586774 10.1161/CIR.0000000000000625 PMC7403606

[19] Mast N , El‐Darzi N , Li Y , Pikuleva IA . Quantitative characterizations of the cholesterol‐related pathways in the retina and brain of hamsters. J Lipid Res. 2023;64:100401. 37330011 10.1016/j.jlr.2023.100401 PMC10394389

[20] El‐Darzi N , Mast N , Li Y , Pikuleva IA . APOB100 transgenic mice exemplify how the systemic circulation content may affect the retina without altering retinal cholesterol input. Cell Mol Life Sci. 2024;81:52. 38253888 10.1007/s00018-023-05056-4 PMC10803575

[21] van Leeuwen EM , Emri E , Merle BMJ , et al. A new perspective on lipid research in age‐related macular degeneration. Prog Retin Eye Res. 2018;67:56‐86. 29729972 10.1016/j.preteyeres.2018.04.006

[22] Camus MC , Chapman MJ , Forgez P , Laplaud PM . Distribution and characterization of the serum lipoproteins and apoproteins in the mouse, *Mus musculus* . J Lipid Res. 1983;24:1210‐1228. 6631247

[23] Goulinet S , Chapman MJ . Plasma lipoproteins in the golden Syrian hamster (*Mesocricetus auratus*): heterogeneity of apoB‐ and apoA‐I‐containing particles. J Lipid Res. 1993;34:943‐959. 8354960

[24] Runkle EA , Antonetti DA . The blood‐retinal barrier: structure and functional significance. In: Nag S , ed. The Blood‐Brain and Other Neural Barriers: Reviews and Protocols. Humana Press; 2011:133‐148. 10.1007/978-1-60761-938-3_5 21082369

[25] Elner VM . Retinal pigment epithelial acid lipase activity and lipoprotein receptors: effects of dietary omega‐3 fatty acids. Trans Am Ophthalmol Soc. 2002;100:301‐338. 12545699 PMC1358968

[26] Tserentsoodol N , Sztein J , Campos M , et al. Uptake of cholesterol by the retina occurs primarily via a low density lipoprotein receptor‐mediated process. Mol Vis. 2006;12:1306‐1318. 17110914

[27] Duncan KG , Bailey KR , Kane JP , Schwartz DM . Human retinal pigment epithelial cells express scavenger receptors BI and BII. Biochem Biophys Res Commun. 2002;292:1017‐1022. 11944916 10.1006/bbrc.2002.6756

[28] Duncan KG , Hosseini K , Bailey KR , et al. Expression of reverse cholesterol transport proteins ATP‐binding cassette A1 (ABCA1) and scavenger receptor BI (SR‐BI) in the retina and retinal pigment epithelium. Br J Ophthalmol. 2009;93:1116‐1120. 19304587 10.1136/bjo.2008.144006 PMC3541028

[29] Hayes KC , Lindsey S , Stephan ZF , Brecker D . Retinal pigment epithelium possesses both LDL and scavenger receptor activity. Invest Ophthalmol Vis Sci. 1989;30:225‐232. 2536645

[30] Zheng W , Reem RE , Omarova S , et al. Spatial distribution of the pathways of cholesterol homeostasis in human retina. PLoS One. 2012;7:e37926. 22629470 10.1371/journal.pone.0037926 PMC3358296

[31] Picard E , Houssier M , Bujold K , et al. CD36 plays an important role in the clearance of oxLDL and associated age‐dependent sub‐retinal deposits. Aging. 2010;2:981‐989. 21098885 10.18632/aging.100218 PMC3034186

[32] Ryeom SW , Sparrow JR , Silverstein RL . CD36 participates in the phagocytosis of rod outer segments by retinal pigment epithelium. J Cell Sci. 1996;109(Pt 2):387‐395. 8838662 10.1242/jcs.109.2.387

[33] Houssier M , Raoul W , Lavalette S , et al. CD36 deficiency leads to choroidal involution via COX2 down‐regulation in rodents. PLoS Med. 2008;5:e39. 18288886 10.1371/journal.pmed.0050039 PMC2245984

[34] Lin JB , Mast N , Bederman IR , et al. Cholesterol in mouse retina originates primarily from in situ de novo biosynthesis. J Lipid Res. 2016;57:258‐264. 26630912 10.1194/jlr.M064469 PMC4727421

[35] Malek G , Li CM , Guidry C , Medeiros NE , Curcio CA . Apolipoprotein B in cholesterol‐containing drusen and basal deposits of human eyes with age‐related maculopathy. Am J Pathol. 2003;162:413‐425. 12547700 10.1016/S0002-9440(10)63836-9 PMC1851166

[36] Li CM , Chung BH , Presley JB , et al. Lipoprotein‐like particles and cholesteryl esters in human Bruch's membrane: initial characterization. Invest Ophthalmol Vis Sci. 2005;46:2576‐2586. 15980251 10.1167/iovs.05-0034

[37] Li CM , Presley JB , Zhang X , et al. Retina expresses microsomal triglyceride transfer protein: implications for age‐related maculopathy. J Lipid Res. 2005;46:628‐640. 15654125 10.1194/jlr.M400428-JLR200

[38] Wang L , Li CM , Rudolf M , et al. Lipoprotein particles of intraocular origin in human Bruch membrane: an unusual lipid profile. Invest Ophthalmol Vis Sci. 2009;50:870‐877. 18806290 10.1167/iovs.08-2376 PMC2692837

[39] Curcio CA , Johnson M , Huang JD , Rudolf M . Aging, age‐related macular degeneration, and the response‐to‐retention of apolipoprotein B‐containing lipoproteins. Prog Retin Eye Res. 2009;28:393‐422. 19698799 10.1016/j.preteyeres.2009.08.001 PMC4319375

[40] Curcio CA , Johnson M , Rudolf M , Huang JD . The oil spill in ageing Bruch membrane. Br J Ophthalmol. 2011;95:1638‐1645. 21890786 10.1136/bjophthalmol-2011-300344 PMC3633599

[41] Curcio CA . Soft drusen in age‐related macular degeneration: biology and targeting via the oil spill strategies. Invest Ophthalmol Vis Sci. 2018;59:AMD160‐AMD181. 30357336 10.1167/iovs.18-24882 PMC6733535

[42] Curcio CA . Antecedents of soft drusen, the specific deposits of age‐related macular degeneration, in the biology of human macula. Invest Ophthalmol Vis Sci. 2018;59:AMD182‐AMD194. 30357337 10.1167/iovs.18-24883 PMC6733529

[43] Curcio CA , Millican CL . Basal linear deposit and large drusen are specific for early age‐related maculopathy. Arch Ophthalmol. 1999;117:329‐339. 10088810 10.1001/archopht.117.3.329

[44] Wong WL , Su X , Li X , et al. Global prevalence of age‐related macular degeneration and disease burden projection for 2020 and 2040: a systematic review and meta‐analysis. Lancet Glob Health. 2014;2:e106‐e116. 25104651 10.1016/S2214-109X(13)70145-1

[45] Tserentsoodol N , Gordiyenko NV , Pascual I , Lee JW , Fliesler SJ , Rodriguez IR . Intraretinal lipid transport is dependent on high density lipoprotein‐like particles and class B scavenger receptors. Mol Vis. 2006;12:1319‐1333. 17110915

[46] Bojanic DD , Tarr PT , Gale GD , et al. Differential expression and function of ABCG1 and ABCG4 during development and aging. J Lipid Res. 2010;51:169‐181. 19633360 10.1194/jlr.M900250-JLR200 PMC2789777

[47] Ananth S , Gnana‐Prakasam JP , Bhutia YD , et al. Regulation of the cholesterol efflux transporters ABCA1 and ABCG1 in retina in hemochromatosis and by the endogenous siderophore 2,5‐dihydroxybenzoic acid. Biochim Biophys Acta. 2014;1842:603‐612. 24462739 10.1016/j.bbadis.2014.01.010 PMC4289134

[48] Zheng W , Mast N , Saadane A , Pikuleva IA . Pathways of cholesterol homeostasis in mouse retina responsive to dietary and pharmacologic treatments. J Lipid Res. 2015;56:81‐97. 25293590 10.1194/jlr.M053439 PMC4274074

[49] Ban N , Lee TJ , Sene A , et al. Disrupted cholesterol metabolism promotes age‐related photoreceptor neurodegeneration. J Lipid Res. 2018;59:1414‐1423. 29946056 10.1194/jlr.M084442 PMC6071770

[50] Ban N , Lee TJ , Sene A , et al. Impaired monocyte cholesterol clearance initiates age‐related retinal degeneration and vision loss. JCI Insight. 2018;3(17):e120824. doi:10.1172/jci.insight.120824 30185655 PMC6171801

[51] Storti F , Klee K , Todorova V , et al. Impaired ABCA1/ABCG1‐mediated lipid efflux in the mouse retinal pigment epithelium (RPE) leads to retinal degeneration. elife. 2019;8:e45100. doi:10.7554/eLife.45100 30864945 PMC6435327

[52] Lin JB , Halawa OA , Husain D , Miller JW , Vavvas DG . Dyslipidemia in age‐related macular degeneration. Eye. 2022;36:312‐318. 35017697 10.1038/s41433-021-01780-y PMC8807842

[53] El‐Darzi N , Mast N , Dailey B , et al. Characterizations of hamster retina as a model for studies of retinal cholesterol homeostasis. Biology. 2021;10:1003. 34681102 10.3390/biology10101003 PMC8533155

[54] Dutta S , Sengupta P . Men and mice: relating their ages. Life Sci. 2016;152:244‐248. 26596563 10.1016/j.lfs.2015.10.025

[55] Dutta S , Sengupta P . Age of laboratory hamster and human: drawing the connexion. Biomed Pharmacol J. 2019;12(1):49‐56. doi:10.13005/bpj/1612

[56] Flower RW , Hochheimer BF . Clinical infrared absorption angiography of the choroid. Am J Ophthalmol. 1972;73:458‐459. 4622382 10.1016/0002-9394(72)90079-7

[57] Yoneya S , Noyori K . Improved visualization of the choroidal circulation with indocyanine green angiography. Arch Ophthalmol. 1993;111:1165‐1166. 7993387 10.1001/archopht.1993.01090090015002

[58] Mordon S , Devoisselle JM , Soulie‐Begu S , Desmettre T . Indocyanine green: physicochemical factors affecting its fluorescence in vivo. Microvasc Res. 1998;55:146‐152. 9521889 10.1006/mvre.1998.2068

[59] Chen L , Zhang X , Li M , Liao N , Wen F . Age‐related scattered hypofluorescent spots on late‐phase indocyanine green angiography as precursor lesions of polypoidal choroidal vasculopathy. Invest Ophthalmol Vis Sci. 2019;60:2102‐2109. 31095678 10.1167/iovs.19-26968

[60] Chang AA , Morse LS , Handa JT , et al. Histologic localization of indocyanine green dye in aging primate and human ocular tissues with clinical angiographic correlation. Ophthalmology. 1998;105:1060‐1068. 9627657 10.1016/S0161-6420(98)96008-0

[61] Jung H , Liu J , Liu T , et al. Longitudinal adaptive optics fluorescence microscopy reveals cellular mosaicism in patients. JCI Insight. 2019;4(6):e124904. doi:10.1172/jci.insight.124904 30895942 PMC6483009

[62] Chen L , Yang P , Curcio CA . Visualizing lipid behind the retina in aging and age‐related macular degeneration, via indocyanine green angiography (ASHS‐LIA). Eye (Lond). 2022;36:1735‐1746. 35314773 10.1038/s41433-022-02016-3 PMC9391351

[63] Li J , Aguilera N , Liu T , et al. Structural integrity of retinal pigment epithelial cells in eyes with age‐related scattered hypofluorescent spots on late phase indocyanine green angiography (ASHS‐LIA). Eye (Lond). 2023;37:377‐378. 36115884 10.1038/s41433-022-02232-x PMC9873905

[64] Chen L , Zhang X , Liu B , Mi L , Wen F . Age‐related scattered hypofluorescent spots on late‐phase indocyanine green angiography: the multimodal imaging and relevant factors. Clin Experiment Ophthalmol. 2018;46:908‐915. 29675907 10.1111/ceo.13306 PMC6282543

[65] Chang AA , Zhu M , Billson F . The interaction of indocyanine green with human retinal pigment epithelium. Invest Ophthalmol Vis Sci. 2005;46:1463‐1467. 15790916 10.1167/iovs.04-0825

[66] Tam J , Liu J , Dubra A , Fariss R . In vivo imaging of the human retinal pigment epithelial mosaic using adaptive optics enhanced indocyanine green ophthalmoscopy. Invest Ophthalmol Vis Sci. 2016;57:4376‐4384. 27564519 10.1167/iovs.16-19503 PMC5015921

[67] Li K , Wong DK , Luk FS , Kim RY , Raffai RL . Isolation of plasma lipoproteins as a source of extracellular RNA. In: Patel T , ed. Extracellular RNA: Methods and Protocols. Springer; 2018:139‐153. 10.1007/978-1-4939-7652-2_11 PMC6476197 29388141

[68] Saadane A , Mast N , Charvet CD , et al. Retinal and nonocular abnormalities in *Cyp27a1* ^−/−^ *Cyp46a1* ^−/−^ mice with dysfunctional metabolism of cholesterol. Am J Pathol. 2014;184:2403‐2419. 25065682 10.1016/j.ajpath.2014.05.024 PMC4188134

[69] Zhao Y , Marcel YL . Serum albumin is a significant intermediate in cholesterol transfer between cells and lipoproteins. Biochemistry. 1996;35:7174‐7180. 8679545 10.1021/bi952242v

[70] Sankaranarayanan S , de la Llera‐Moya M , Drazul‐Schrader D , Phillips MC , Kellner‐Weibel G , Rothblat GH . Serum albumin acts as a shuttle to enhance cholesterol efflux from cells. J Lipid Res. 2013;54:671‐676. 23288948 10.1194/jlr.M031336 PMC3617942

[71] Lai SJ , Ohkawa R , Horiuchi Y , Kubota T , Tozuka M . Red blood cells participate in reverse cholesterol transport by mediating cholesterol efflux of high‐density lipoprotein and apolipoprotein A‐I from THP‐1 macrophages. Biol Chem. 2019;400:1593‐1602. 31188743 10.1515/hsz-2019-0244

[72] Zoanni B , Brioschi M , Mallia A , et al. Novel insights about albumin in cardiovascular diseases: focus on heart failure. Mass Spectrom Rev. 2023;42:1113‐1128. 34747521 10.1002/mas.21743

[73] Expert Panel on Detection, Evaluation, and Treatment of High Blood Cholesterol in Adults . Executive summary of the third report of the National Cholesterol Education Program (NCEP) expert panel on detection, evaluation, and treatment of high blood cholesterol in adults (adult treatment panel III). JAMA. 2001;285:2486‐2497. 11368702 10.1001/jama.285.19.2486

[74] National Cholesterol Education Program (NCEP) Expert Panel on Detection, Evaluation, and Treatment of High Blood Cholesterol in Adults (Adult Treatment Panel III) . Third report of the National Cholesterol Education Program (NCEP) expert panel on detection, evaluation, and treatment of high blood cholesterol in adults (adult treatment panel III) final report. Circulation. 2002;106:3143‐3421. 12485966

[75] Morrisett JD , Kim HS , Patsch JR , Datta SK , Trentin JJ . Genetic susceptibility and resistance to diet‐induced atherosclerosis and hyperlipoproteinemia. Arteriosclerosis. 1982;2:312‐324. 7115205 10.1161/01.atv.2.4.312

[76] Cherrick GR , Stein SW , Leevy CM , Davidson CS . Indocyanine green: observations on its physical properties, plasma decay, and hepatic extraction. J Clin Invest. 1960;39:592‐600. 13809697 10.1172/JCI104072 PMC293343

[77] Baker KJ . Binding of sulfobromophthalein (BSP) sodium and indocyanine green (ICG) by plasma alpha‐1 lipoproteins. Proc Soc Exp Biol Med. 1966;122:957‐963. 5918158 10.3181/00379727-122-31299

[78] Janecki J , Krawcynski J . Labeling with indocyanine green of serum protein from normal persons and patients with acute viral hepatitis. Clin Chem. 1970;16:1008‐1011. 5481559

[79] Kamisaka K , Yatsuji Y , Yamada H , Kameda H . The binding of indocyanine green and other organic anions to serum proteins in liver diseases. Clin Chim Acta. 1974;53:255‐264. 4366883 10.1016/0009-8981(74)90107-7

[80] Yoneya S , Saito T , Komatsu Y , Koyama I , Takahashi K , Duvoll‐Young J . Binding properties of indocyanine green in human blood. Invest Ophthalmol Vis Sci. 1998;39:1286‐1290. 9620093

[81] Desmettre T , Devoisselle JM , Mordon S . Fluorescence properties and metabolic features of indocyanine green (ICG) as related to angiography. Surv Ophthalmol. 2000;45:15‐27. 10946079 10.1016/s0039-6257(00)00123-5

[82] Hehnly H , Stamnes M . Regulating cytoskeleton‐based vesicle motility. FEBS Lett. 2007;581:2112‐2118. 17335816 10.1016/j.febslet.2007.01.094 PMC1974873

[83] Yu H . HDL and scavenger receptor class B type I (SRBI). In: Zheng L , ed. HDL Metabolism and Diseases. Springer Nature; 2022:79‐93. 10.1007/978-981-19-1592-5_6 35575922

[84] Valle M . Pyruvate carboxylase, structure and function. Subcell Biochem. 2017;83:291‐322. 28271481 10.1007/978-3-319-46503-6_11

[85] Bisaccia F , Indiveri C , Palmieri F . Purification of reconstitutively active α‐oxoglutarate carrier from pig heart mitochondria. Biochim Biophys Acta Bioenerg. 1985;810:362‐369. 10.1016/0005-2728(85)90222-1 4063354

[86] Fiermonte G , Palmieri L , Todisco S , Agrimi G , Palmieri F , Walker JE . Identification of the mitochondrial glutamate transporter. Bacterial expression, reconstitution, functional characterization, and tissue distribution of two human isoforms. J Biol Chem. 2002;277:19289‐19294. 11897791 10.1074/jbc.M201572200

[87] Esteban‐Amo MJ , Jiménez‐Cuadrado P , Serrano‐Lorenzo P , de la Fuente M , Simarro M . Succinate dehydrogenase and human disease: novel insights into a well‐known enzyme. Biomedicine. 2024;12:2050. 10.3390/biomedicines12092050 PMC11429145 39335562

[88] Thompson K , Majd H , Dallabona C , et al. Recurrent de novo dominant mutations in SLC25A4 cause severe early‐onset mitochondrial disease and loss of mitochondrial DNA copy number. Am J Hum Genet. 2016;99:860‐876. 27693233 10.1016/j.ajhg.2016.08.014 PMC5065686

[89] Klingenberg M . The ADP and ATP transport in mitochondria and its carrier. Biochim Biophys Acta Biomembr. 2008;1778:1978‐2021. 10.1016/j.bbamem.2008.04.011 18510943

[90] Yoshida M , Muneyuki E , Hisabori T . ATP synthase—a marvellous rotary engine of the cell. Nat Rev Mol Cell Biol. 2001;2:669‐677. 11533724 10.1038/35089509

[91] Tauchmannová K , Pecinová A , Houštěk J , Mráček T . Variability of clinical phenotypes caused by isolated defects of mitochondrial ATP synthase. Physiol Res. 2024;73:S243‐S278. 39016153 10.33549/physiolres.935407 PMC11412354

[92] Elkholi R , Abraham‐Enachescu I , Trotta AP , et al. MDM2 integrates cellular respiration and apoptotic signaling through NDUFS1 and the mitochondrial network. Mol Cell. 2019;74:452‐465.e457. 30879903 10.1016/j.molcel.2019.02.012 PMC6499641

[93] Agip AA , Blaza JN , Bridges HR , et al. Cryo‐EM structures of complex I from mouse heart mitochondria in two biochemically defined states. Nat Struct Mol Biol. 2018;25:548‐556. 29915388 10.1038/s41594-018-0073-1 PMC6054875

[94] Baertling F , Sánchez‐Caballero L , van den Brand MAM , et al. NDUFAF4 variants are associated with Leigh syndrome and cause a specific mitochondrial complex I assembly defect. Eur J Hum Genet. 2017;25:1273‐1277. 28853723 10.1038/ejhg.2017.133 PMC5643967

[95] Rhein VF , Carroll J , Ding S , Fearnley IM , Walker JE . NDUFAF5 hydroxylates NDUFS7 at an early stage in the assembly of human complex I. J Biol Chem. 2016;291:14851‐14860. 27226634 10.1074/jbc.M116.734970 PMC4938201

[96] Jamin A , Wiebe MS . Barrier to autointegration factor (BANF1): interwoven roles in nuclear structure, genome integrity, innate immunity, stress responses and progeria. Curr Opin Cell Biol. 2015;34:61‐68. 26072104 10.1016/j.ceb.2015.05.006 PMC4522355

[97] Seal RL , Denny P , Bruford EA , et al. A standardized nomenclature for mammalian histone genes. Epigenetics Chromatin. 2022;15:34. 36180920 10.1186/s13072-022-00467-2 PMC9526256

[98] Phelan ML , Sif S , Narlikar GJ , Kingston RE . Reconstitution of a core chromatin remodeling complex from SWI/SNF subunits. Mol Cell. 1999;3:247‐253. 10078207 10.1016/s1097-2765(00)80315-9

[99] Abdul Rehman SA , Kristariyanto YA , Choi SY , et al. MINDY‐1 is a member of an evolutionarily conserved and structurally distinct new family of deubiquitinating enzymes. Mol Cell. 2016;63:146‐155. 27292798 10.1016/j.molcel.2016.05.009 PMC4942677

[100] David Y , Ziv T , Admon A , Navon A . The E2 ubiquitin‐conjugating enzymes direct polyubiquitination to preferred lysines. J Biol Chem. 2010;285:8595‐8604. 20061386 10.1074/jbc.M109.089003 PMC2838281

[101] Nguyen H , Kettenbach AN . Substrate and phosphorylation site selection by phosphoprotein phosphatases. Trends Biochem Sci. 2023;48:713‐725. 37173206 10.1016/j.tibs.2023.04.004 PMC10523993

[102] Allen PB , Ouimet CC , Greengard P . Spinophilin, a novel protein phosphatase 1 binding protein localized to dendritic spines. Proc Natl Acad Sci USA. 1997;94:9956‐9961. 9275233 10.1073/pnas.94.18.9956 PMC23308

[103] Hediger MA , Clemencon B , Burrier RE , Bruford EA . The ABCs of membrane transporters in health and disease (SLC series): introduction. Mol Asp Med. 2013;34:95‐107. 10.1016/j.mam.2012.12.009 PMC3853582 23506860

[104] Truebestein L , Leonard TA . Coiled‐coils: the long and short of it. Bioessays. 2016;38:903‐916. 27492088 10.1002/bies.201600062 PMC5082667

[105] Lupas AN , Gruber M . The structure of α‐helical coiled coils. Advances in Protein Chemistry. Vol 70. Academic Press; 2005:37‐38. 15837513 10.1016/S0065-3233(05)70003-6

[106] Ola MS , Hosoya K , LaNoue KF . Influence of insulin on glutamine synthetase in the Müller glial cells of retina. Metab Brain Dis. 2011;26:195‐202. 21626103 10.1007/s11011-011-9245-y

[107] Lieth E , LaNoue KF , Berkich DA , et al. Nitrogen shuttling between neurons and glial cells during glutamate synthesis. J Neurochem. 2001;76:1712‐1723. 11259489 10.1046/j.1471-4159.2001.00156.x

[108] Wong‐Riley MT . Energy metabolism of the visual system. Eye Brain. 2010;2:99‐116. 23226947 10.2147/EB.S9078 PMC3515641

[109] Cohen LH , Noell WK . Glucose catabolism of rabbit retina before and after development of visual function. J Neurochem. 1960;5:253‐276. 13810977 10.1111/j.1471-4159.1960.tb13363.x

[110] Joyal JS , Sun Y , Gantner ML , et al. Retinal lipid and glucose metabolism dictates angiogenesis through the lipid sensor Ffar1. Nat Med. 2016;22:439‐445. 26974308 10.1038/nm.4059 PMC4823176

[111] Gasnier B . The SLC32 transporter, a key protein for the synaptic release of inhibitory amino acids. Pflugers Arch. 2004;447:756‐759. 12750892 10.1007/s00424-003-1091-2

[112] Davanger S , Ottersen OP , Storm‐Mathisen J . Glutamate, GABA, and glycine in the human retina: an immunocytochemical investigation. J Comp Neurol. 1991;311:483‐494. 1684589 10.1002/cne.903110404

[113] Pikuleva IA , Curcio CA . Cholesterol in the retina: the best is yet to come. Prog Retin Eye Res. 2014;41:64‐89. 24704580 10.1016/j.preteyeres.2014.03.002 PMC4058366

[114] Feher J , Kovacs I , Artico M , Cavallotti C , Papale A , Balacco Gabrieli C . Mitochondrial alterations of retinal pigment epithelium in age‐related macular degeneration. Neurobiol Aging. 2006;27:983‐993. 15979212 10.1016/j.neurobiolaging.2005.05.012

[115] Terluk MR , Kapphahn RJ , Soukup LM , et al. Investigating mitochondria as a target for treating age‐related macular degeneration. J Neurosci. 2015;35:7304‐7311. 25948278 10.1523/JNEUROSCI.0190-15.2015 PMC4420790

[116] Fisher CR , Ferrington DA . Perspective on AMD pathobiology: a bioenergetic crisis in the RPE. Invest Ophthalmol Vis Sci. 2018;59:AMD41‐AMD47. 30025108 10.1167/iovs.18-24289 PMC5989860

