# Supplementary material for: Dietary effects on the retina of hamsters
Source: FASEB J. 2025 Mar 18;39(6):e70451. doi: 10.1096/fj.202403390R (PMC11917192; doi:10.1096/fj.202403390R)
Supplement: Supplementary file 1 — Figure S1. [file FSB2-39-e70451-s002.pdf]

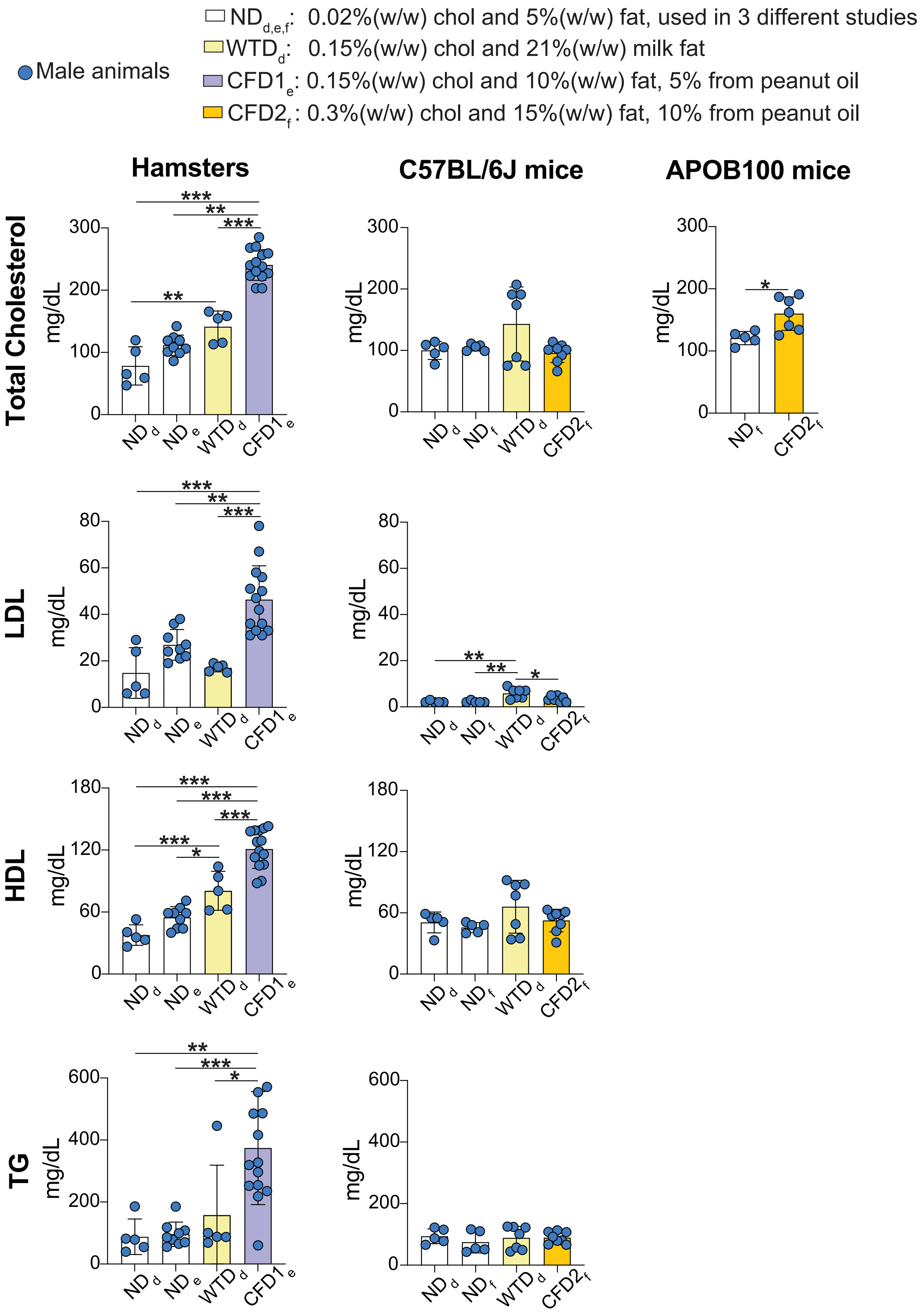

Supplemental Figure 1. A comparision of dietary effects on plasma lipid profile in the same species. The diets and studies are the same as in Table 1. Data represent the mean ± SD of the measurements in individual animals (n=5-14 per group). For hamsters and C57BL/6J mice, statistical significance was assessed by one-way ANOVA with Tukey's multiple comparison test. For APOB100 mice, a two-tailed unpaired Student’s t test was used. $^{*}P \leq 0.05$; $^{**}P \leq 0.01$; $^{***}P \leq 0.001$. CFD, cholesterol- and fat-enriched diet; ND, normal diet; and WTD, Western-type diet.
