# Supplementary material for: Dietary effects on the retina of hamsters
Source: FASEB J. 2025 Mar 18;39(6):e70451. doi: 10.1096/fj.202403390R (PMC11917192; doi:10.1096/fj.202403390R)
Supplement: Supplementary file 2 — Figure S2. [file FSB2-39-e70451-s001.pdf]

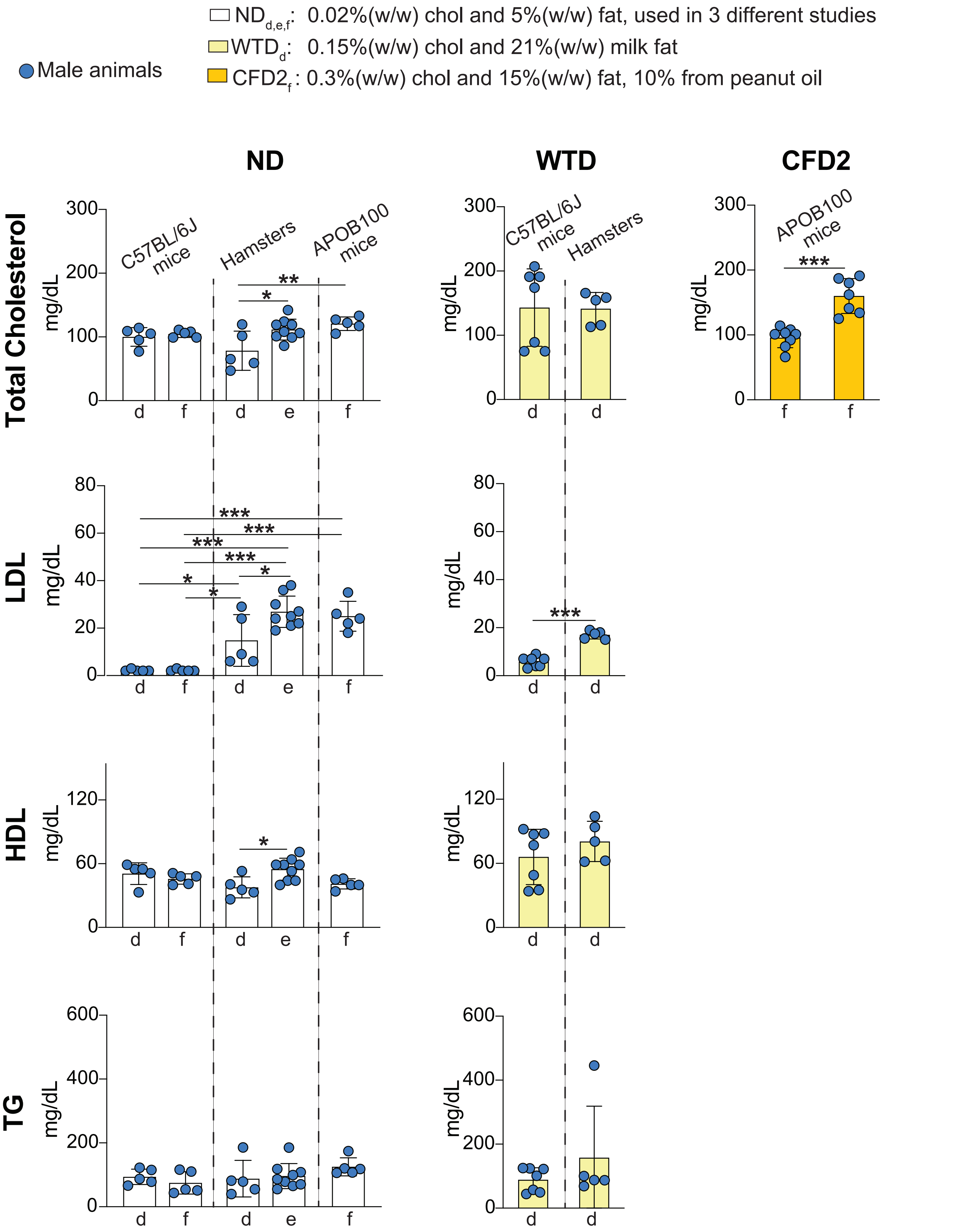

Supplemental Figure 2. A comparision of the same diet effect on plasma lipid profile in different species. The diets and studies are the same as in Table 1. Data represent the mean ± SD of the measurements in individual animals (n=5-14 per group). For normal diet (ND), statistical significance was assessed by one-way ANOVA with Tukey's multiple comparison test. For Western-type diet (WTD) and cholesterol- and fat-enriched diet 2 (CFD2), a two-tailed unpaired Student's t test was used. *$P \leq 0.05$; **$P \leq 0.01$; ***$P \leq 0.001$.
